# A cysteine-based molecular code informs collagen C-propeptide assembly

**DOI:** 10.1038/s41467-018-06185-2

**Published:** 2018-10-11

**Authors:** Andrew S. DiChiara, Rasia C. Li, Patreece H. Suen, Azade S. Hosseini, Rebecca J. Taylor, Alexander F. Weickhardt, Diya Malhotra, Darrell R. McCaslin, Matthew D. Shoulders

**Affiliations:** 1 Department of Chemistry, Massachusetts Institute of Technology, 77 Massachusetts Avenue, Cambridge, MA 02139 USA; 2 Department of Biochemistry, University of Wisconsin, 433 Babcock Drive, Madison, WI 53706 USA

## Abstract

Fundamental questions regarding collagen biosynthesis, especially with respect to the molecular origins of homotrimeric versus heterotrimeric assembly, remain unanswered. Here, we demonstrate that the presence or absence of a single cysteine in type-I collagen’s C-propeptide domain is a key factor governing the ability of a given collagen polypeptide to stably homotrimerize. We also identify a critical role for Ca^2+^ in non-covalent collagen C-propeptide trimerization, thereby priming the protein for disulfide-mediated covalent immortalization. The resulting cysteine-based code for stable assembly provides a molecular model that can be used to predict, *a priori*, the identity of not just collagen homotrimers, but also naturally occurring 2:1 and 1:1:1 heterotrimers. Moreover, the code applies across all of the sequence-diverse fibrillar collagens. These results provide new insight into how evolution leverages disulfide networks to fine-tune protein assembly, and will inform the ongoing development of designer proteins that assemble into specific oligomeric forms.

## Abstract (web summary)

Collagen proteins assemble into trimers from distinct monomers with high specificity, yet the molecular basis for this specificity remains unclear. Here the authors demonstrate the crucial role of conserved C-terminal domain cysteine residues and calcium in homotrimeric procollagen assembly.

## Introduction

Construction of the functionally diverse collagenous extracellular matrices, including bone, skin, cartilage, tendon, and beyond, is among the most remarkable biological supramolecular assembly processes^1,2^. The formation of these distinctive tissues begins with and depends upon proper folding and assembly of procollagen triple helices in the endoplasmic reticulum (ER). The abundant fibrillar collagens consist of the classical triple-helical domain of collagen^3^, appended at each terminus by globular N-propeptide and C-propeptide domains (termed N-Pro and C-Pro) that are proteolytically removed post secretion. The C-Pro domain, which is cysteine-rich, *N*-glycosylated, and ~30 kDa in typical fibrillar collagens, plays a particularly important role in collagen assembly, as collagen folding begins at the C-terminus^4,5^. Thus, the C-Pro domain is responsible not only for initiating triple-helix folding, but also for strand selection and for establishing correct triple-helix register to promote the formation of structurally sound fibrils and tissues^6^.

**Fig. 1:**
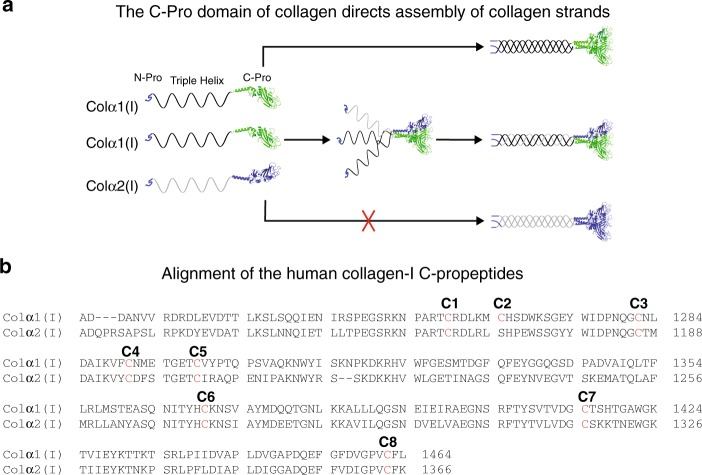
C-Pro domain-mediated assembly of collagen type-I. **a** Schematic representation of collagen-I assembly. Two strands of Colα1(I) and one strand of Colα2(I) typically assemble into heterotrimers, a process that is initiated by the respective C-Pro domains. Colα1(I) is also known to form homotrimers, whereas Colα2(I) does not homotrimerize and only forms heterotrimers. The crystal structure of a homotrimeric C-Pro domain is used here for illustration purposes (PDBID 5K31), with each collagen-I C-Pro domain differentially colored to demonstrate possible assembly schematics^16^. **b** Alignment of the Colα1(I) and Colα2(I) C-Pro domains highlights high sequence similarity. The cysteine network is numbered from C1 to C8 in the N-terminal to C-terminal direction, with each cysteine residue colored in red

Elucidating the molecular mechanisms that guide collagen assembly is central to our understanding of collagen biosynthesis. In humans, there are 28 known collagen types, but >45 genetically distinctive collagen strands^1^. The excess of collagen strands relative to collagen types means that some collagens form 2:1 or even 1:1:1 heterotrimers. For example, type-II and type-III collagen are homotrimeric; the most abundant collagen, type-I, is typically a 2:1 heterotrimer of two Colα1(I) chains and one Colα2(I) chain (Fig. 1a); and collagen type-V can form a 1:1:1 heterotrimer of Colα1(V), Colα2(V), and Colα3(V)^1^. Homotrimeric versus heterotrimeric triple helices have different stability, altered propensities for extracellular supramolecular assembly, and customized functions and binding partners^7^. The unique features and functionalities of collagen heterotrimers have rendered formation of defined synthetic collagen heterotrimers a major goal of peptide and protein engineers^8–15^. However, attaining and maintaining defined triple-helix compositions and proper register in short collagen-like peptides capable of biomimetic supramolecular assembly continues to present significant challenges.

**Fig. 2:**
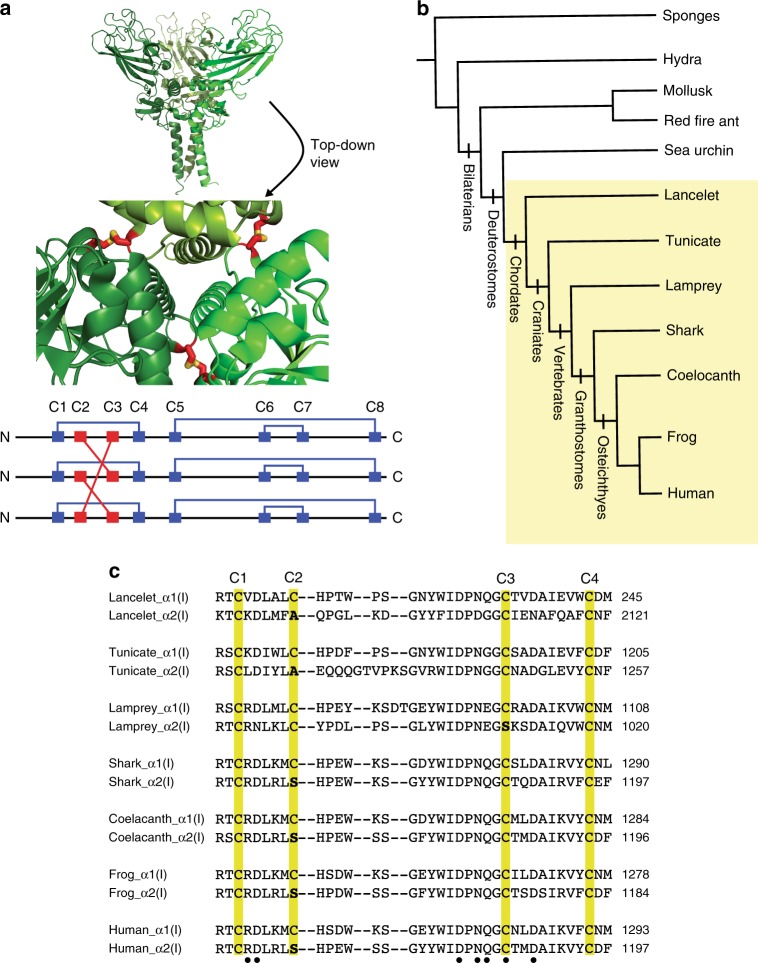
Phylogenetic analysis of the cysteine network of the C-Pro domain of collagen-I. **a** Crystal structure of C-Proα1(I) (top; PDBID 5K31) oriented to display the interstrand disulfide bonds (shown in stick mode) between the C2 and C3 residues (numbered as in Fig. 1b) of neighboring polypeptides that covalently stitch the identical subunits together^16^. Each subunit is colored in a different shade of green to facilitate visualization. **b** Cladogram illustrating organismal evolution of animals. Each clade of the tree is listed at each branch point, with the organisms likely to have heterotrimeric collagen-I highlighted by the yellow box. **c** Alignment of ancient collagen-I C-Pro sequences, beginning with the earliest likely emergence of heterotrimeric collagen-I (chordates). Conserved cysteine residues are highlighted in yellow, with the cysteine number indicated at the top of the alignment. Sites where C2 (or C3) are not conserved are bolded. Conserved amino acids involved in the Ca^2+^ coordination network are marked with a “•” below the alignment (see also Fig. 4 for a representative image of the Ca^2+^ coordination). Collagen C-Pro domains were aligned using Clustal Omega

Despite two recent high-resolution structures of collagen C-Pro domain homotrimers^16,17^, we still lack structural information for collagen C-Pro domain heterotrimers. Consequently, the molecular features of the C-Pro domain that guide a given collagen strand to form homotrimers versus heterotrimers remain poorly understood. In each case of the heterotrimeric collagen types, at least one of the genetically encoded polypeptides is known to be capable of forming homotrimers, whereas the others are only capable of forming heterotrimers. For example, Colα1(I) can stably homotrimerize, whereas Colα2(I) cannot (Fig. 1a)^1,18,19^. Alignment of human Colα1(I) and Colα2(I) reveals strong similarities in the C-propeptide region, with 62% sequence identity and 78% similarity. One striking difference is that the homotrimer-forming Colα1(I) C-Pro has eight cysteine residues (Fig. 1b; annotated as C1 through C8 with numbering beginning from the most N-terminal cysteine residue in the C-Pro domain), while the heterotrimer-only-forming Colα2(I) C-Pro has only seven cysteine residues. In Colα2(I), the second cysteine residue present in Colα1(I) (C2) is replaced by a serine. The crystal structure of assembled Colα1(I) C-Pro homotrimers revealed that C1–C4, C5–C8, and C6–C7 form intrastrand disulfide bonds, whereas C2 and C3 participate in interstrand disulfide bonds that covalently stitch the three C-Pro domains together (Fig. 2a)^16^.

Given the strong sequence similarity between the Colα1(I) and Colα2(I) C-Pro domains, an appealing hypothesis is that the absence of C2 is a fundamental factor abrogating the ability to form stable homotrimers^20^. Early studies evaluating this hypothesis relied on the use of a collagen mini-gene system, comprised of the N-propeptide and C-propeptide of Colα2(I) or Colα1(III) C-Pro appended on short, ~200-amino acid triple-helical domains (note that Colα1(III) is a homotrimerizing collagen and that Colα1(I) was not studied)^21–23^. That early, seminal work surprisingly indicated little or no role for the presence or absence of C2 in controlling collagen homotrimerization versus heterotrimerization. In addition to evaluating whether C2 controls trimerization propensities, in other work using the collagen mini-gene system a 23-amino acid discontinuous sequence in the C-Pro domain, termed the chain recognition sequence, was identified and proposed to regulate collagen type-specific assembly^23^. The crystal structure of the Colα1(III) C-Pro homotrimer is consistent with a role for the chain recognition sequence in type-specific assembly^17^, likely discouraging other collagen types from assembling with collagen-III in cells that synthesize more than one collagen type. However, the relative lack of specific inter-subunit interactions involving the chain recognition sequence in the Colα1(I) C-Pro homotrimer structure hints that this sequence may not be sufficient on its own to specify collagen type-specific assembly^16^. More recently, additional amino acids at the interface between individual subunits in Colα1(I) homotrimers or 2:1 Colα1(I):Colα2(I) heterotrimers were proposed to be relevant^16^. While these studies provide insights into the collagen type-specific assembly problem, for the even more fundamental question of whether a given collagen strand can form homotrimers versus heterotrimers, it remains the case that no single amino acid substitution has been identified to date that can swap homotrimerizing versus heterotrimerizing propensities.

Here, we build on phylogenetic analyses and the recent crystal structures of homotrimeric C-Pro domains to revisit the possibility that a cysteine-based molecular code plays a central role in defining collagen trimerization propensities. Via a comprehensive suite of biochemical and biophysical studies, we demonstrate the critical function of covalent disulfide bond formation in defining stable C-Pro domain assembly. We also discover the high innate propensity of all collagen-I C-Pro domains to non-covalently trimerize with themselves and each other, resulting in a new mechanistic model for collagen C-Pro trimerization that explains previously contradictory data. Cumulatively, these results unmask the central role of the cysteine code in guiding stable collagen C-Pro assembly, and reveal how nature leverages the identity of a single atom in a 30 kDa protein domain to guide the assembly of a complex multimeric protein.

## Results

### Phylogenetic analysis of the C-Pro cysteine network

We began by aligning collagen C-Pro domains from distantly related species across the animal kingdom to track the appearance of each factor that has been proposed to play a key role in collagen assembly through evolutionary history. Our goal was to identify a conserved factor that emerged near the same point as collagen-I heterotrimers, which likely evolved no later than the last common ancestor of all chordates (see Fig. 2b for a cladogram). Previous modeling based on the crystal structure of C-Proα1(I) suggested that Lys1248 and Glu1249 (numbering based on the full-length procollagen α1(I) sequence) in the chain recognition sequence of human C-Proα2(I) may form heterotrimer-stabilizing salt bridges with the adjacent C-Proα1(I) in a collagen-I heterotrimer^16^. However, Lys1248 probably did not fix until osteichthyes, and a negatively charged amino acid at position 1249 probably did not evolve until tetrapods (see Fig. 2b and Supplementary Fig. 1). Moreover, only Asp1347, but not Asp1344, the salt bridge-forming amino acids in C-Proα1(I), is present in sharks, one of the early-diverging groups that later lost bone (Supplementary Fig. 1). Therefore, Asp1344 probably was absent in the last common ancestor of gnathostomes and did not fix until osteichthyes. Other amino acids in C-Proα2(I), including Arg1165 and Lys1366, have also been proposed to form salt bridges with the chain recognition sequence in C-Proα1(I)^16^, but evidence that Arg1165 and Lys1366 impact homotrimerization of C-Proα2(I) is lacking. Beyond these specific amino acids, the chain recognition sequence itself likely did not appear until gnathostomes (Supplementary Fig. 1). Thus, this sequence appears to be a recently evolved vertebrate mechanism of chain selection that developed only when the problem of type-specific collagen assembly became significantly complicated by the presence of many collagen types in a single organism. An alternative explanation is clearly required to account for the homotrimerizing versus heterotrimerizing propensities of collagen strands.

In contrast to the relatively recent evolution of the chain recognition sequence, analysis of distantly related collagens reveals that the distinctive cysteine substitution patterns of Colα1(I) and Colα2(I) C-Pro domains is much more highly conserved (C1 through C4 shown in Fig. 2c; see also the full alignment shown in Supplementary Fig. 1)^24^. Indeed, the pattern of one strand containing eight cysteine residues and the other containing seven cysteine residues emerged along with the chordates, which were likely the earliest organisms to display collagen-I heterotrimers, and has been maintained since that time throughout distantly related groups of chordates.

### Evaluation of the role of interstrand disulfide bonds

The strong conservation of the cysteine pattern (Fig. 2c) across chordates, a group of animals with highly divergent body plans, appears to provide compelling support for the hypothesis that the presence or absence of specific Cys residues critically regulates the ability of collagen-I strands to homotrimerize versus heterotrimerize. Therefore, we were motivated to revisit the conclusion from prior research that re-introduction of C2 in C-Proα2(I) does not allow the protein to stably homotrimerize^21,23^.

**Fig. 3:**
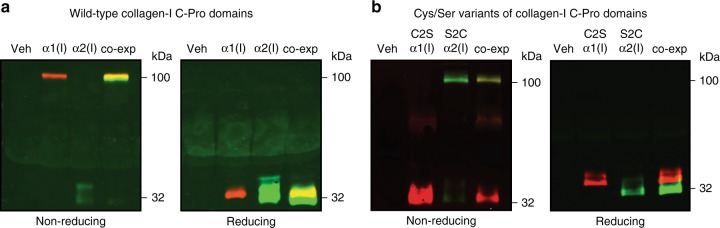
The presence or absence of a single cysteine residue defines the capacity of collagen-I C-Pro domains to form stable, disulfide-linked homotrimers. **a** Immunoblot analysis of individually expressed wild-type, HA-tagged C-Proα1(I) (red) and wild-type, FLAG-tagged C-Proα2(I) (green) proteins under non-reducing and reducing conditions showing that wild-type C-Proα1(I) forms a disulfide-linked homotrimer, whereas wild-type C-Proα2(I) does not, recapitulating the known disulfide-dependent assembly patterns of full-length Colα1(I) and Colα2(I). Co-expression of wild-type C-Proα1(I) and wild-type C-Proα2(I) rescues wild-type C-Proα2(I) into a heterotrimer, as shown by the yellow color indicating red and green overlap. **b** Immunoblot analysis of individually expressed HA-tagged Cys1265Ser (C2S) C-Proα1(I) (red) and FLAG-tagged Ser1169Cys (S2C) C-Proα2(I) (green) proteins under non-reducing and reducing conditions showing that the serine variant of C-Proα1(I) is no longer able to form a disulfide-linked homotrimer. In contrast, the cysteine variant of C-Proα2(I) is able to form disulfide-linked homotrimers. Co-expression of C2S C-Proα1(I) and S2C C-Proα2(I) rescues C2S C-Proα1(I) into a heterotrimer, as shown by the yellow color indicating red and green overlap

We began by creating plasmids for expression of hemagglutinin (HA)-tagged C-Proα1(I) and FLAG-tagged C-Proα2(I) in human cells. The distinctive antibody epitopes were included to simplify differential detection by immunoblotting. We incorporated a preprotrypsin signal sequence to target the proteins to the ER for folding and subsequent secretion. We found that, when expressed alone in human embryonic kidney 293 (HEK293) cells, both C-Proα1(I) (as previously observed^16^) and C-Proα2(I) were robustly secreted. Denaturing sodium dodecyl sulfate-polyacrylamide gel electrophoresis (SDS-PAGE) immunoblot analysis of the media under non-reducing conditions demonstrated the expected assembly patterns for these proteins. C-Proα1(I) migrated as a stable, disulfide-linked homotrimer (Fig. 3a), whereas C-Proα2(I) migrated as a monomer. Both C-Proα1(I) and C-Proα2(I) migrated as monomers under reducing SDS-PAGE conditions (Fig. 3a). These results recapitulate the known ability of full-length Colα1(I) to form disulfide-linked homotrimers and the known inability of full-length Colα2(I) to do the same. Furthermore, co-expression of C-Proα1(I) and C-Proα2(I) rescued monomeric C-Proα2(I) into a disulfide-linked heterotrimer with C-Proα1(I) (Fig. 3a; shown by the yellow overlap on a non-reducing gel upon co-expression). Thus, consistent with prior work^16^, these biochemically amenable constructs provide a valid model system to examine the molecular code for collagen assembly.

Next, we tested the hypothesis that the disulfide-linked trimerization propensities of the Colα1(I) and Colα2(I) C-Pro domains can be transposed simply by replacing C2 with a serine residue in C-Proα1(I) and restoring the cysteine residue at the C2 position in C-Proα2(I) (Fig. 1b). We observed that both these variants were robustly secreted from HEK293 cells. Strikingly, the Cys1265Ser variant of C-Proα1(I) (hereafter referred to as C2S) migrated as a monomer on a non-reducing SDS-PAGE gel (Fig. 3b). In contrast, the Ser1169Cys (hereafter referred to as S2C) variant of C-Proα2(I) migrated as a disulfide-linked homotrimer (Fig. 3b). Both variants migrated as monomers under reducing conditions. Remarkably, as also observed for the co-expression of the wild-type C-Proα(I) domains in Fig. 3a, co-expression of C2S C-Proα1(I) with S2C C-Proα2(I) rescued monomeric C2S C-Proα1(I) into a disulfide-linked heterotrimer (Fig. 3b; shown by the yellow overlap upon co-expression). Thus, altering just one amino acid, and indeed changing the identity of only a single atom in the entire ~30 kDa C-Pro domain, is sufficient to transpose the trimerization propensities of C-Proα1(I) and C-Proα2(I).

### The role of Ca^2+^ in non-covalent collagen C-Pro assembly

**Fig. 4:**
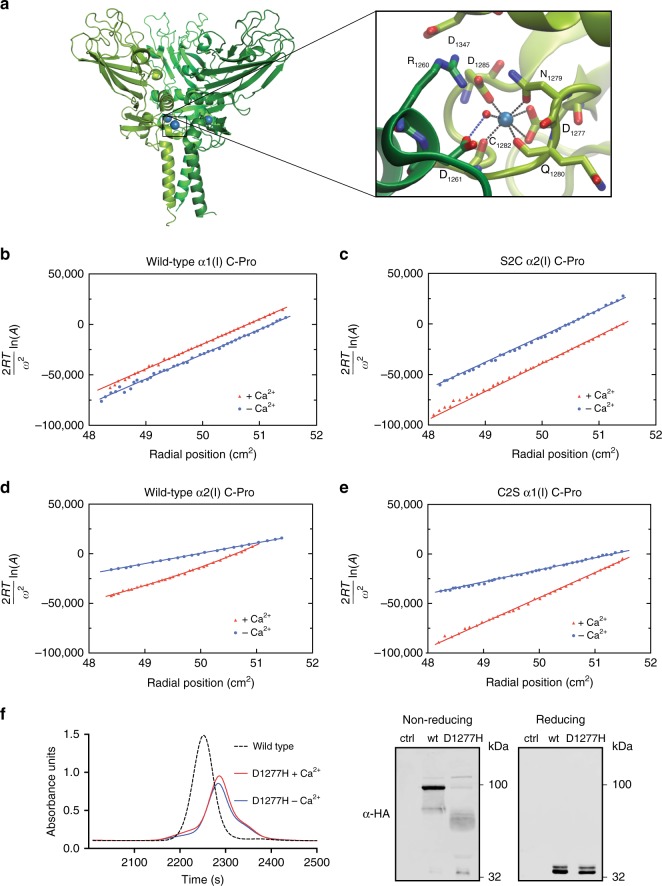
The essential role of Ca^2+^ in templating non-covalent C-Pro assembly. **a** Crystal structure of C-Proα1(I) (PDBID 5K31)^16^, highlighting the presence of a Ca^2+^ ion at each subunit interface of the homotrimer. Each subunit is colored in a different shade of green with Ca^2+^ ions in blue. Inset: residues involved in binding Ca^2+^. In **b**–**c**, the slopes are directly proportional to the weight average molecular weight at each point. **b** Sedimentation equilibrium data showing that wild-type C-Proα1(I) is best fit as a single homotrimeric species in the absence of Ca^2+^ (blue) and in the presence of Ca^2+^ (red). The best fit is shown as a solid line. **c** Sedimentation equilibrium data showing that S2C C-Proα2(I) is best fit as a single homotrimeric species both in the absence of Ca^2+^ (blue) and in the presence of Ca^2+^ (red). The best fit is shown as a solid line in the corresponding color. In **d**–**e**, the slopes, which vary at each radial position, are directly proportional to the weight average molecular weight for the total protein concentration at that position. **d** Sedimentation equilibrium data for wild-type C-Proα2(I) in the absence of Ca^2+^ are best fit as a monomer–dimer equilibrium (primarily monomer; see Supplementary Fig. 4) with an association constant sufficiently small to be approximated as a single species of intermediate weight-average molecular weight, with no evidence of a trimeric species, as shown by the best fit in blue. In the presence of 0.5 mM Ca^2+^ (red), the data are best fit as a monomer–trimer equilibrium (primarily trimer; see Supplementary Fig. 4). **e** Sedimentation equilibrium data for C2S C-Proα1(I) in the absence of calcium are best fit as a monomer–dimer equilibrium (primarily monomer; see Supplementary Fig. 5) with an association constant sufficiently small to be approximated as a single species of intermediate weight-average molecular weight, with no evidence of trimeric species, as shown by the best fit in blue. In the presence of 0.5 mM Ca^2+^ (red), the data are best fit as a monomer–trimer equilibrium (primarily trimer; see Supplementary Fig. 5). Only every third data point is shown (**b**–**e**). **f** Size exclusion chromatography analysis of Asp1277His C-Proα1(I) in the presence and absence of Ca^2+^, as compared to wild-type C-Proα1(I), reveals the inability of this Ca^2+^-binding variant to assemble into a homotrimer, despite the presence of both C2 and C3 (left). Immunoblot analysis under non-reducing conditions show the predominant species is a dimer, with other oligomeric products also present (right)

The data in Fig. 3 show that the presence or absence of C2 in the collagen-I C-Pro domain is a defining feature controlling the ability to homotrimerize in a disulfide-dependent manner, consistent with the phylogenetic analyses in Fig. 2. Critically, however, results derived from SDS-PAGE gels do not address the innate ability of these C-Pro domains to trimerize independent of disulfide bond formation, as the analyses are necessarily performed under denaturing instead of native conditions. Furthermore, our observations in Fig. 3 conflict with prior work on the C2 variant of a Colα2(I) mini-gene consisting of the N-Pro domain, a short triple-helical domain, and the C-Pro domain^21,23^. In those prior studies, disulfide-linked homotrimers were not observed for S2C C-Proα2(I) on a non-reducing SDS-PAGE gel. Moreover, the short triple-helical domain was reported to be sensitive to proteolysis, suggesting that a triple helix was not formed^23^. Notably, the model proteins in those early studies were expressed in a cell-free, rabbit reticulocyte expression system, either in the presence or absence of canine pancreatic microsomes. The final step before inducing expression of a protein of interest in such a system is treatment with EDTA or EGTA to complex Ca^2+^, thereby inactivating the Ca^2+^-dependent nuclease used to degrade endogenous mRNAs^25^. Examination of the crystal structures of C-Pro homotrimers suggests that the resulting Ca^2+^ depletion during synthesis and folding may be confounding, as Ca^2+^ ions are bound at the interfaces between individual subunits of the trimer (Fig. 4a)^16,17^.

To address this conflict and better understand the innate trimerization propensities of collagen-I C-Pro domains, we purified milligram quantities of His-tagged versions of all four C-Proα(I) variants, cleaving the His tag to yield unmodified C-Pro domains in the final purification step (see Supplementary Fig. 2). We note that liquid chromatography-mass spectrometry analysis of both the purified C-Proα1(I) and S2C C-Proα2(I) homotrimers revealed the expected disulfide linkages based on the existing crystal structures of homotrimeric C-Pro domains^16,17^ upon proteolysis of the oxidized and denatured samples, suggesting that the purified homotrimers are both properly folded (Supplementary Tables 1 and 2).

Next, we obtained sedimentation equilibrium data for each purified C-Pro protein variant in the presence or absence of 0.5 mM Ca^2+^ (ER Ca^2+^ concentrations may be as high as 2 mM^26^). In Fig. 4b–e, representative data are shown wherein the slopes of the tangents at any point are proportional to the weight-average molecular weight of the species present at that point. The data for each variant were evaluated by various one-species and two-species models^27^.

We found that, for wild-type C-Proα1(I) and S2C C-Proα2(I), the data (best fits shown; Fig. 4b–c and Supplementary Fig. 3) were consistent with a single homotrimeric species with no evidence of smaller species present in solution. The results were independent of the presence of Ca^2+^ in the buffer for these interstrand disulfide-linked homotrimers. The Ca^2+^ independence of these results was expected, as both C-Proα1(I) and S2C C-Proα2(I) contain C2 and C3 and were initially folded and covalently immortalized as homotrimers inside the Ca^2+^-rich, intact ER.

In contrast, for wild-type C-Proα2(I) the data revealed a clear Ca^2+^-dependence. In Ca^2+^-lacking buffer, wild-type C-Proα2(I) was best described as a monomer–dimer equilibrium with an association constant small enough to be approximated as a single species of intermediate molecular weight (Fig. 4d, blue). At the speeds and concentrations used, we saw no direct evidence for any trimeric species in the absence of Ca^2+^. On the other hand, in the presence of 0.5 mM Ca^2+^, the plot for wild-type C-Proα2(I) (Fig. 4d, red) was curved, indicating the existence of multiple species. The data were best described as an equilibrium mixture of monomer and trimer (best fit shown in red). Examples of the gradient fits are provided in Supplementary Fig. 4.

Similar trends were observed for C2S C-Proα1(I). In the absence of Ca^2+^, C2S C-Proα1(I), like wild-type C-Proα2(I), was best fit as a single species of intermediate molecular weight, indicating a monomer–dimer equilibrium with a small association constant (Fig. 4e, blue). Again, no direct evidence for any trimeric species was observed in the absence of Ca^2+^. In the presence of Ca^2+^ (Fig. 4e, red), the data for C2S C-Proα1(I) were best described as an equilibrium mixture of monomer and trimer (best fit shown in red). Examples of the gradient fits are provided in Supplementary Fig. 5.

These sedimentation equilibrium studies resolve the conflict between our data and prior work, while also revealing an important new feature of collagen C-Pro assembly. In the presence of biologically relevant Ca^2+^ concentrations, both wild-type C-Proα2(I) and S2C C-Proα1(I) can unexpectedly homotrimerize. On the other hand, under Ca^2+^-depleted conditions, such as those caused by EDTA or EGTA treatment of rabbit reticulocyte lysates prior to inducing C-Pro translation, non-covalent homotrimerization by both wild-type C-Proα2(I) and S2C C-Proα1(I) is minimal. As non-covalent trimerization is an essential first step before formation of stable, disulfide-linked homotrimers, the absence of Ca^2+^ in rabbit reticulocyte lysate likely prevented observation of the disulfide-linked homotrimerization of S2C C-Proα2(I) mini-genes^21^, leading to the conclusion in those studies that interstrand disulfide bond-forming cysteines do not critically regulate homotrimerization.

To further evaluate this conclusion, we next asked whether Ca^2+^ is required for non-covalently templating assembly of even the natively homotrimerizing, C2-containing wild-type C-Proα1(I) protein in cells. Asp1277His is an osteogenesis imperfecta-causing amino acid substitution in Colα1(I) that reduces trimer formation. Because Asp1277 is one of the amino acids involved in Ca^2+^ binding^28^, we anticipated that this substitution might prevent Ca^2+^-templated non-covalent assembly of C-Proα1(I) and that disulfide-linked homotrimers might no longer form. Indeed, size exclusion chromatography (SEC) analysis of Asp1277His C-Proα1(I) demonstrated that this variant does not form disulfide-linked homotrimers, instead forming a mixture of dimers and monomers (Fig. 4f). A non-reducing, SDS-PAGE gel immunoblot further confirmed that, unlike wild-type C-Proα1(I), Asp1277His C-Proα1(I) does not form stable, disulfide-linked homotrimers to any significant extent. These results lend further credence to our conclusion that Ca^2+^-mediated non-covalent assembly is an essential first step that precedes the covalent immortalization of stable C-Proα(I) trimeric species.

### Assembly of disulfide-linked C-Proα(I) heterotrimers

**Fig. 5:**
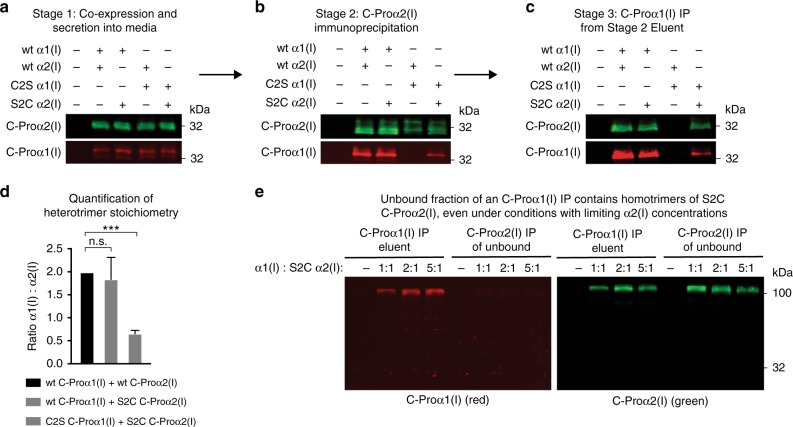
Co-expression analysis of heterotrimeric assembly products. This figure is supplemented with arrows from **a** to **b** and from **b** to **c** to indicate that the samples generated from each stage were then used in the subsequent analysis. Both wild-type and C2S C-Proα1(I) were tagged with HA. Both wild-type and S2C C-Proα2(I) were tagged with FLAG. All C-Proα1(I) signal was detected using an antibody raised against C-Proα1(I) (shown in red). All C-Proα2(I) signal was detected using an antibody raised against the FLAG epitope (shown in green). **a** Stage 1: Media harvested from cells transfected with the indicated combinations of C-Proα1(I) and C-Proα2(I) was immunoblotted for the presence of each construct on a reducing SDS-PAGE gel. **b** Stage 2: Immunoprecipitation of C-Proα2(I) from the Stage 1 media samples (**a**) under non-reducing, Ca^2+^-depleted conditions. Immunoprecipitated samples were immunoblotted for the presence of each construct on a reducing SDS-PAGE gel. **c** Stage 3: Final step in the purification of heterotrimers via immunoprecipitation of C-Proα1(I) from the eluent obtained in Stage 2 (**b**). Immunoprecipitated samples were immunoblotted for the presence of each construct on a reducing SDS-PAGE gel. **d** Quantification of the C-Proα1(I):C-Proα2(I) ratio of the purified heterotrimers obtained in Stage 3 (**c**). The bar chart shows the average ratio across four biological replicates. ***p value <0.001 as determined by a *t* test. Error bars indicate standard deviation from the mean for each sample. **e** Co-immunoprecipitation experiments on heterotrimers formed when wild-type C-Proα1(I) and S2C C-Proα2(I) were co-expressed at ratios of 1:1, 2:1, and 5:1. The conditioned media were first subjected to an HA immunoprecipitation to extract all C-Proα1(I)-containing species, and the supernatant (unbound fraction) was then subjected to a FLAG immunoprecipitation to purify any remaining S2C C-Proα2(I). Elutions from the FLAG immunoprecipitation were analyzed on a non-reducing, SDS-PAGE gel. n.s. = not significant

We next used a sequential immunoprecipitation and immunoblotting experimental strategy to more closely examine the composition of the stable trimeric species formed upon co-expression of assorted C-Proα(I) variants. As illustrated in Fig. 5, we co-expressed wild-type C-Proα1(I) with wild-type C-Proα2(I), wild-type C-Proα1(I) with S2C C-Proα2(I), C2S C-Proα1(I) with wild-type C-Proα2(I), or C2S C-Proα1(I) with S2C C-Proα2(I). All the C-Proα1(I) constructs were HA-tagged and all the C-Proα2(I) constructs were FLAG-tagged in these experiments, such that we could immunopurify and then employ immunoblotting to differentially isolate and identify the proteins. We used immunoblotting of reducing SDS-PAGE gels to probe the C-Pro domains present in the solution at each of three stages: Stage 1, directly upon secretion into media (Fig. 5a); Stage 2, after immunoprecipitating C-Proα2(I) using anti-FLAG beads under non-reducing conditions in the presence of EDTA to deplete Ca^2+^ (Fig. 5b); and Stage 3, after immunoprecipitating stable C-Proα1(I)-containing heterotrimers from the Stage 2 eluent using HA beads under the same conditions (Fig. 5c).

Consistent with our results in Fig. 3, co-expression of wild-type C-Proα1(I) with wild-type C-Proα2(I) resulted in detection of both species at all stages of the experiment (Fig. 4a–c), with the purified 2:1 heterotrimer presumably the only species present after Stage 3 (we confirmed the 2:1 C-Proα1(I):C-Proα2(I) stoichiometry of this heterotrimer using our purified C-Proα1(I) and C-Proα2(I) to create standard curves). Co-expression of wild-type C-Proα1(I) with S2C C-Proα2(I) also resulted in detection of both species at all stages of the experiment (Fig. 5a–c), indicating that S2C C-Proα2(I) was able to form disulfide-linked heterotrimers with wild-type C-Proα1(I). Notably, the stoichiometry of the purified heterotrimers in Fig. 5c was again ~2:1 C-Proα1(I):C-Proα2(I) (Fig. 5d), as expected. In contrast, co-expression of C2S C-Proα1(I) with wild-type C-Proα2(I) did not co-immunoprecipitate any C-Proα1(I) after Stage 2 (Fig. 5b), consistent with an inability to form stable, disulfide-linked heterotrimers when both C-Proα1(I) and C-Proα2(I) lacked C2. Finally, co-expression of C2S C-Proα1(I) with S2C C-Proα2(I) again resulted in detection of both species at all stages of the experiment (Fig. 5a–c), consistent with the formation of a stable, disulfide-linked heterotrimer. Notably, the stoichiometry of the purified heterotrimer was now 1:2 C-Proα1(I):C-Proα2(I), further demonstrating that swapping which C-Pro domain has C2 can completely function swap their roles.

We next asked whether S2C C-Proα2(I) can form disulfide-linked homotrimers even in the presence of excess wild-type C-Proα1(I). We analyzed the S2C C-Proα2(I) assembly products remaining in conditioned media after an HA immunoprecipitation to remove any wild-type C-Proα1(I)-containing species. We found that S2C C-Proα2(I) does indeed form stable, disulfide-linked homotrimers, even in the presence of a large excess of wild-type C-Proα1(I) (Fig. 5e). The presence of disulfide-linked homotrimers of S2C C-Proα2(I), even in the context of a wild-type C-Proα1(I) co-expression experiment, likely explains why it is so critical for C-Proα2(I) to lack C2. Specifically, stable homotrimerization of S2C C-Proα2(I) is possible even when abundant wild-type C-Proα1(I) is present. Apparently, the only mechanism to prevent stable C-Proα2(I) homotrimerization is to remove C2.

### 1:1:1 Collagen heterotrimers

The observation that a single amino acid substitution can swap the disulfide-linked oligomerization propensities of the Colα1(I) and Colα2(I) C-Pro domains is provocative, suggesting that the cysteine pattern is key for guiding the formation of 2:1 collagen heterotrimers. If the interstrand disulfide linkages are indeed critical, we should also be able to build a model that predicts the sequences required to favor formation of a 1:1:1 collagen heterotrimer. Importantly, 1:1:1 collagen heterotrimers are biologically relevant in multiple domains of life (e.g., collagen type-V in humans^29^ and collagen type-I in rainbow trout^30^ and zebrafish^31^).

**Fig. 6:**
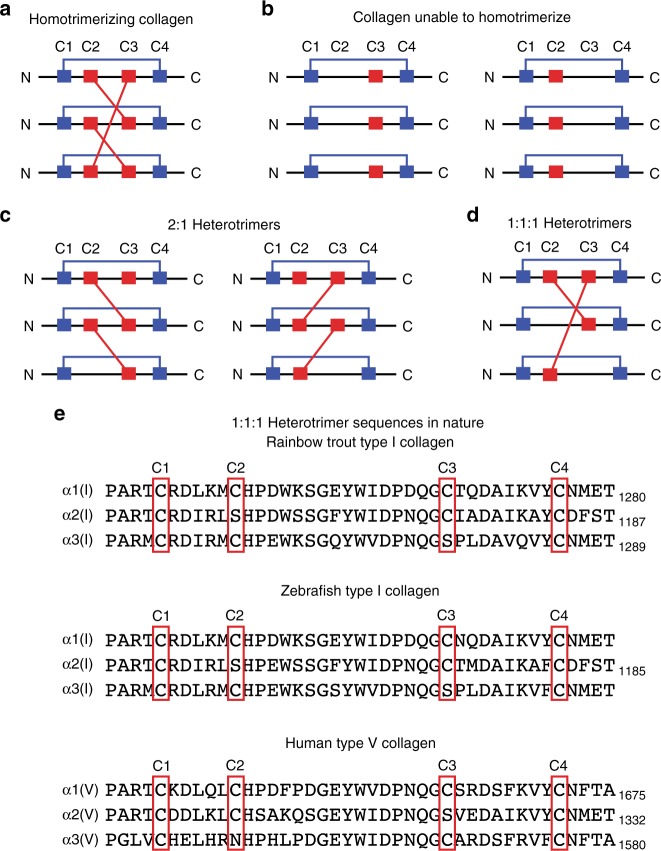
*A priori* prediction of collagen trimerization propensities based on the C2/C3 pattern (C1 through C4 shown). **a** Known disulfide-bonding network for a homotrimeric collagen strand with C2 and C3 intact, as revealed by the crystal structures of C-Proα1(I) and C-Proα1(III) homotrimers^16, 17^. **b** Predicted disulfide-bonding network for a C-Pro domain lacking a cysteine residue in position 2 or 3. Although intrastrand disulfide bonds likely form, interstrand disulfide bonds cannot form in the absence of C2 or C3. **c** Predicted disulfide-bonding network for a collagen heterotrimer that requires 2:1 assembly. The accuracy of this predicted disulfide-bonding pattern in 2:1 heterotrimers is supported by previous work modeling the replacement of a single C-Proα1(I) subunit in the homotrimer crystal structure with a C-Proα2(I) monomer^16^. Note the single free cysteine in the assembled protein owing to the presence of an odd number of cysteine residues. **d** Predicted disulfide-bonding network for a collagen heterotrimer that requires 1:1:1 assembly, illustrating the minimum requirements for stable 1:1:1 heterotrimer formation, which are one strand having cysteine residues at positions 2 and 3, one only at position 2, and one only at position 3. **e** Alignment of rainbow trout and zebrafish collagen-I C-Pro domains (C1–C4 only shown). The expected cysteine pattern for a 1:1:1 homotrimer in **d** is present in both instances where collagen-I has three α strands in nature and in human collagen type-V, which all form 1:1:1 heterotrimers. Note that in all images the potentially interstrand disulfide-forming cysteines (C2 and C3) are colored red, while the intrastrand disulfide-forming cysteines are colored blue

Our model here is based on the disulfide-bonding pattern observed in the crystal structures of the C-Proα1(I) and C-Proα1(III) homotrimers. In those structures, C1–C4, C5–C8, and C6–C7 all form intrastrand disulfide bonds following the pattern in Fig. 2a. Assuming this pattern is conserved across all the fibrillar collagen C-Pro domains, only C2 and C3 remain to form the interstrand disulfides that covalently immortalize C-Pro trimers, and thereby regulate heterotrimer formation. In a C-Pro homotrimer, C2 of each subunit forms a disulfide with C3 of the neighboring subunit (Fig. 6a). C-Pro domains that lack C2 (or C3, in principle) cannot form disulfide-linked homotrimers, as a disulfide-forming partner for the single cysteine present is not available (Fig. 6b). In a disulfide-linked 2:1 heterotrimer, assuming the assembled C-Pro structure is similar to that of a homotrimer (a hypothesis that is supported by low-resolution small-angle X-ray scattering data on the collagen-I heterotrimer^16^), the strand lacking C2 (or C3) can only be present once, and the strand present twice must have both C2 and C3 (Fig. 6c; this is the situation observed in human collagen-I). The result is a single free cysteine present in a 2:1 C-Pro heterotrimer. Finally, to form a stable, disulfide-linked 1:1:1 heterotrimer, one strand must have both C2 and C3, one strand should lack C2, and the third strand should lack C3 (Fig. 6d).

Remarkably, this final predicted pattern is exactly what is observed in known 1:1:1 heterotrimers of fibrillar collagens (Fig. 6e). Rainbow trout collagen-I, zebrafish collagen-I, and human collagen-V all have one genetic strand with both C2 and C3, one lacking C2, and one lacking C3. We note that the cysteine code likely also provides insight into the relative topologies of the 1:1:1 heterotrimers that form in these species. Given the difference in cysteine patterns between Colα2(I) and Colα3(I) of rainbow trout and zebrafish versus Colα2(V) and Colα3(V) in humans, the α2 and α3 chains are likely in different orientations relative to the α1 chain, which maintains a cysteine residue in both the C2 and C3 positions (see Supplementary Fig. 6). In summary, it is likely that the Cys code guides not just 2:1 but also 1:1:1 heterotrimerization, further highlighting its fundamental role as a critical regulatory nexus for collagen trimerization and emphasizing the need for covalent immortalization to stabilize collagen heterotrimers for triple-helix formation.

### Cysteines inform assembly of all fibrillar collagens

**Fig. 7:**
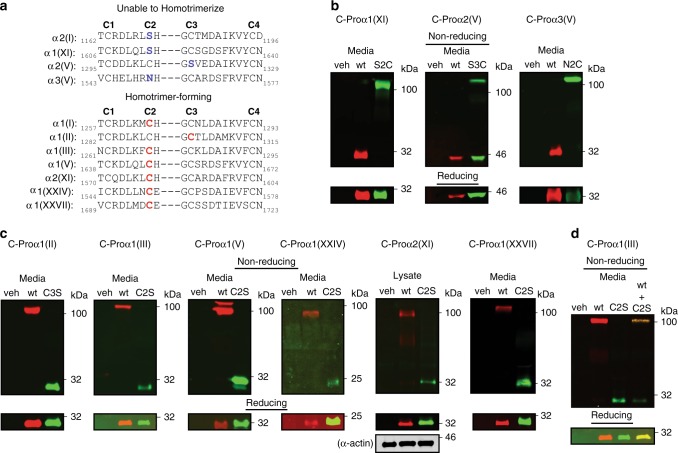
The cysteine-based code for collagen C-Pro assembly is generalizable across the fibrillar collagens. **a** Alignment of the interstrand disulfide-bonding region of the human fibrillar collagen C-propeptides highlights that C-Pro domains known to homotrimerize contain all four conserved cysteine residues, whereas C-Pro domains known to only form heterotrimers lack a single cysteine at C2 or C3. The residue colored red in each protein sequence corresponds to the mutated residue analyzed in **b**, **c**. Amino acid numbering was derived from the corresponding full-length procollagens. **b** Immunoblot analyses of individually expressed wild-type fibrillar collagen C-Pro domains known to only form heterotrimers. Assembly was analyzed under non-reducing and reducing conditions. Wild-type variants (HA-tagged; red) all migrated as monomers, while all variants in which the missing cysteine residue was re-introduced (FLAG-tagged; green) gained the ability to homotrimerize in a disulfide-dependent manner. **c** Immunoblot analysis of individually expressed wild-type fibrillar collagen C-Pro domains known to have the capacity to homotrimerize. Assembly was analyzed under non-reducing and reducing conditions. Wild-type variants (HA-tagged; red) all migrated as disulfide-dependent homotrimers, while variants in which a single cysteine residue was mutated to serine (FLAG-tagged; green) lost the capacity to form disulfide-linked homotrimers. Note that C-Proα2(XI) was not secreted and was detected only in cell lysate. **d** Co-expression of the wild-type C-Proα1(III) domain with the C2S C-Proα1(III) demonstrates the wild-type protein’s ability to rescue the monomeric C2S variant into a disulfide-linked trimer, shown by the overlap of green and red signal (yellow)

The conserved presence of both C2 and C3 in one collagen type-I gene and the absence of C2 in the second collagen type-I gene throughout the evolutionary tree (Fig. 2c), as well as the observation of the expected cysteine pattern for 1:1:1 heterotrimer formation (Fig. 6e), suggests that the cysteine code may be generalizable across the fibrillar collagens. Indeed, alignment of the C-Pro domains of the human fibrillar collagens reveals that all collagen strands known to form homotrimers consistently maintain both C2 and C3 (Fig. 7a). In contrast, those that are unable to form stable homotrimers and instead must heterotrimerize always lack either C2 or C3 (Fig. 7a).

Building on this observation, to experimentally evaluate the generality of the cysteine code, we created HA-tagged constructs for the expression of wild-type C-Pro domains of all the fibrillar collagens and FLAG-tagged constructs for expression of appropriate variants. We found that all of the wild-type fibrillar C-Pro domains that lack either C2 or C3 migrated as monomers on SDS-PAGE gels (Fig. 7b), just as was observed for wild-type C-Proα2(I) (Fig. 3a). Moreover, re-introduction of the missing cysteine residue always conferred the ability to form disulfide-linked homotrimers. Similarly, all of the wild-type fibrillar C-Pro domains that naturally retain all four cysteine residues and are known to form homotrimers migrated as disulfide-linked homotrimers on SDS-PAGE gels (Fig. 7c). Notably, for all of these C-Pro domains, conversion of C2 or C3 to a serine eliminated the ability to form disulfide-linked homotrimers. Finally, we observed that the monomeric, C2S form of collagen-III’s C-Pro domain could be rescued into a disulfide-linked trimer by co-expression with the wild-type C-Proα(III) containing both C2 and C3 (Fig. 7d). Thus, the cysteine-based molecular code for stable collagen trimerization is conserved across a diverse suite of collagen types.

## Discussion

The lack of C2 (or C3) in the C-Pro domain of fibrillar collagen strands that do not homotrimerize was first noted >30 years ago^20^, leading initially to the proposal that the absence of a single cysteine residue prevents homotrimerization of those collagen strands. Consistent with that hypothesis, as highlighted in Fig. 2, the cysteine code is an ancient pattern that emerged nearly concomitant with the earliest heterotrimerizing collagens, and the pattern is conserved into even recently emerging collagen strands, such as type-XI, type-XXIV, and type-XXVII. Despite this compelling evolutionary evidence, experimental results obtained using collagen mini-genes expressed in rabbit reticulocyte lysates in the presence of canine pancreatic microsomes indicated that re-insertion of C2 in C-Proα2(I) does not allow the protein to homotrimerize^21,23^. This finding prompted an ongoing effort to identify other factors in the C-Pro domain that could account for the ability versus inability of the assorted collagen types to homotrimerize. Such efforts have been fruitful in various ways, leading to the discovery by Bulleid and co-workers^23^ of a 23-amino acid discontinuous chain recognition sequence that contributes to collagen type-specific assembly, as well as to the identification of several charged amino acid residues that may promote incorporation of Colα2(I) into type-I heterotrimers^16^. Any possible role, much less a central role, for the cysteine residues as key regulators of the ability of a given collagen strand to stably homotrimerize has, however, been ignored in the decades since.

The biochemical and biophysical studies reported here provide fresh insights into this longstanding dichotomy. In contrast to prior data obtained using the collagen mini-gene approach^21,23^, our initial experiments were fully consistent with the cysteine code hypothesis. Specifically, we observed on non-reducing SDS-PAGE gels that insertion of C2 in C-Proα2(I) actually does enable disulfide-linked homotrimerization (Fig. 3) of an apparently properly folded C-Proα2(I) domain (Supplementary Tables 1 and 2). Neither insertion of a chain recognition sequence from a homotrimerizing C-Pro domain^23^ nor replacement of charged amino acids at interfaces between individual subunits^16^ is required. Moreover, we found that co-expression of S2C C-Proα2(I) with C2S C-Proα1(I) rescues the latter into a disulfide-linked heterotrimer with a 1:2 ratio of C-Proα1(I):C-Proα2(I) (Fig. 5), reversing the stoichiometry of the native heterotrimer and indicating that simply swapping which C-Pro domain has C2 is sufficient to swap their functions.

Why do our results for the homotrimeric assembly of the C-Proα2(I) domain contradict prior work using Colα2(I) mini-genes? The difference in the model systems used must be considered. We studied the C-Proα2(I) domain in isolation, not fused to any triple-helical domain or N-Pro domain. The mini-gene system is comprised of a short, ~200-amino acid triple-helical domain appended on either end by the C-Pro and N-Pro domains^21^. Incorporation of a short triple-helical domain makes both molecular biology and biochemical analysis more feasible than it is for full-length collagen strands, while still mimicking natural collagen. Thus, on its face, the clever mini-gene system appears more biologically relevant. However, fusion to an abiological, short triple-helical domain (and expression using an in vitro system) may obfuscate the normal role of the C-Pro in initiating collagen trimerization, instead allowing the triple helix itself or the N-Pro domain to drive trimerization in certain cases. Indeed, even much shorter triple-helical sequences are able to assemble in vitro in the complete absence of a C-Pro domain^32^, whereas proper assembly of full-length collagen requires the C-Pro. Differences in the melting temperature of the Colα2(I) versus Colα1(III) mini-triple-helical domains used may also complicate data interpretation. Finally, because the mini-collagens studied were neither purified nor biophysically characterized, the conclusions reached depend entirely on denaturing SDS-PAGE gels and proteolysis experiments. On the other hand, studying the C-Pro domain in isolation may provide an improved model system to understand the factors that control C-Pro homotrimerization versus heterotrimerization propensities. The absence of any other domains that could convolute data interpretation is one potential advantage. Furthermore, two recent high-resolution crystal structures of homotrimeric C-Pro domains show that these constructs fold properly when expressed in human cells. Finally, the C-Pro domains can be purified in mg quantities and high yield for biophysical studies^17^.

The opportunity to perform biophysical studies on purified proteins provided the key insight that we believe explains the discrepancy between our data for wild-type and S2C C-Proα2(I) versus the results from prior work that minimized the importance of the missing cysteine residue. We discovered, using sedimentation equilibrium analysis of C-Pro domains in defined buffer systems (Fig. 4), that the presence of Ca^2+^ is critical to enable non-covalent assembly of collagen-I C-Pro domains. Indeed, we found that even wild-type C-Proα2(I) is able to form a transient homotrimer under these native-like conditions (as is C2S C-Proα1(I)). In contrast, in the absence of Ca^2+^, neither wild-type C-Proα2(I) nor C2S C-Proα1(I) can homotrimerize to any detectable level. These results are consistent with the observation of Ca^2+^ ions bound at subunit interfaces in the high-resolution structures of homotrimeric C-Proα1(I) and C-Proα1(III)^16,17^, and with our discovery that the Ca^2+^-binding defective Asp1277His C-Proα1(I) variant does not form disulfide-linked homotrimers. Importantly, the work using collagen mini-genes was performed in buffers treated with EGTA to chelate Ca^2+^^21,23,25^, likely explaining why S2C C-Proα2(I) did not form a disulfide-linked homotrimer in that setting—the protein was unable to homotrimerize even transiently to template interstrand disulfide bond formation owing to the low Ca^2+^ levels.

**Fig. 8:**
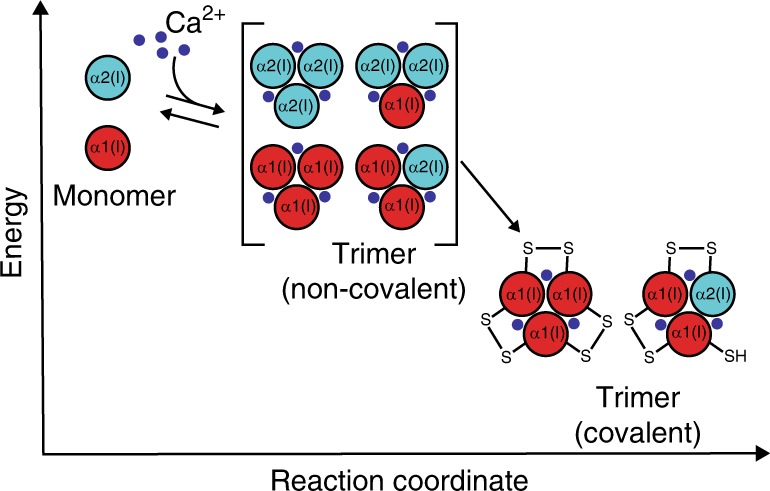
Model reaction coordinate diagram for collagen C-Pro assembly. In the presence of Ca^2+^, collagen C-Pro domains can all transiently trimerize (presumably via initial dimerization; not illustrated here), with the trimers in a dynamic equilibrium with the other states. The non-covalent trimeric assembly products serve as an intermediate to the final covalently immortalized trimers. The transient trimers are bracketed to indicate that their relative energies are not depicted in this plot. Covalent immortalization that is possible only when sufficient cysteine residues are present at positions 2 and 3 to form the requisite disulfides further stabilizes the trimer state, templating collagen triple-helix formation

The discovery that biologically relevant Ca^2+^ concentrations drive transient non-covalent assembly of both C-Proα1(I) and C-Proα2(I) suggests a new model for collagen trimerization (Fig. 8). In a Ca^2+^-rich environment, such as the ER, both wild-type C-Proα1(I) and wild-type C-Proα2(I) can non-covalently homotrimerize to a significant extent, presumably via a monomer → dimer → trimer pathway based on our detection of some dimers in sedimentation equilibrium experiments (Fig. 4). Interstrand disulfide bond formation then provides a thermodynamic sink to stabilize the resulting non-covalent trimers for triple-helix formation. This model explicates the critical importance of a missing cysteine in position 2 of C-Proα2(I) to prevent formation of stable, disulfide-linked homotrimers, instead enabling maintenance of a dynamic equilibrium with the monomer state until a heterotrimer with C-Proα1(I) forms. Indeed, when we co-expressed S2C C-Proα2(I) with even an excess amount of wild-type C-Proα1(I), we still observed formation of disulfide-linked S2C C-Proα2(I) homotrimers (Fig. 5e), highlighting the stability of trimers incorporating C-Proα2(I) and the essentiality of removing the cysteine at position C2 to avoid formation of Colα2(I) homotrimers. The presence of a cysteine in position 3 of C-Proα2(I) is of course also important, because it enables covalent immortalization when the desired non-covalent heterotrimer of C-Proα2(I) with C-Proα1(I) does form.

Further support for the model in Fig. 8 derives from an analysis of the other homotrimerizing and heterotrimerizing fibrillar collagens (we note that non-fibrillar collagens have distinctive sequences and rely on other factors to guide their assembly^33^). The presence of C2 and C3 is conserved across all the homotrimerizing collagen strands, whereas either C2 or C3 is always missing in collagens that are unable to homotrimerize (Fig. 7a). Removal of one of the cysteine residues or insertion of the missing cysteine in all cases swaps the propensity of a given C-Pro domain to form stable, disulfide-linked homotrimers (Fig. 7b, c). The cysteine pattern also explains the propensity of certain collagens to form 1:1:1 heterotrimers (Fig. 6).

Intriguingly, in prior work the wholesale insertion of the C-Proα1(III) chain recognition sequence into C-Proα2(I) did permit non-covalent homotrimerization in the collagen mini-gene system^23^. Although the structural consequences of such a complex chimera remain to be determined, it is noteworthy that the crystal structure of homotrimeric C-Proα1(III) suggests a particularly important role for the chain recognition sequence in stabilizing that particular homotrimer^17^. In contrast, the structure of C-Proα1(I) displays relatively few specific, stabilizing interactions between the chain recognition sequence in neighboring subunits^16^. Bearing in mind the concentration dependence of non-covalent assembly, it seems likely that the chain recognition sequence of type-III collagen is sufficiently stabilizing under conditions of overexpression to eliminate the requirement for an interstrand disulfide bond to promote homotrimerization. This observation highlights that the chain recognition sequence remains an important discovery relevant to the question of type-specific collagen assembly, which we note is not addressed herein. Nonetheless, the conservation of both C2 and C3 in all the homotrimerizing fibrillar collagens indicates that, under native expression conditions, interstrand disulfide bonds are essential to template triple-helix formation. In summary, it appears clear that the presence of a serine instead of a cysteine at position 2 or 3 in the constitutively heterotrimerizing collagens is key for preventing covalent immortalization of transiently formed, non-covalent homotrimers.

The discovery of the innate propensity of collagen C-Pro domains to homotrimerize combined with the discovery that C2 and C3 encode stable homotrimerization versus heterotrimerization indicates that the cysteine code is a critical regulatory nexus controlling collagen assembly. A remaining issue is explaining why collagen strands having both C2 and C3 that are fully capable of forming stable, disulfide-linked homotrimers, such as Colα1(I) and Colα1(V), instead preferentially form heterotrimers with strands lacking C2 or C3 in the biological setting. In collagen type-I, charged residues at the interface between C-Proα1(I) and C-Proα2(I) may favor non-covalent assembly of C-Proα1(I) into heterotrimers rather than homotrimers^16^. In addition, preferential heterotrimerization of Colα1(I) may be mediated by regulated expression and/or assistance from components of the ER’s oxidative protein folding machinery, including protein disulfide isomerases that may assist disulfide shuffling during collagen assembly^34–36^.

In conclusion, our results reveal that a simple cysteine code underpins the homotrimerization versus heterotrimerization propensities of collagen C-Pro domains. Disruptions to cysteine code function are likely to be involved in the pathology of disease-causing mutations in collagen C-Pro domains that result in amino acid substitutions at or near these and other critical cysteine residues^37,38^.

## Methods

### Plasmids

Plasmids encoding the human *Col1A1* and *Col1A2* genes were obtained from Origene. The C-Pro-encoding sequences (from the endogenous C-proteinase cleavage site to the C-terminus of the protein) were PCR-amplified using primers to incorporate the *Not*I and *Eco*RV sites and inserted into pcDNA3.1 vectors encoding the preprotrypsin signal sequence upstream of an HA or FLAG epitope tag, respectively. Variants were created by site-directed mutagenesis using the QuikChange XL II Kit from Agilent Technologies. For purification of recombinant collagen-I C-Pro domains, these vectors were modified to incorporate a cleavable 6×-His tag fused to the C-Pro domain via an HRV-3C protease cleavage site such that the tag could be cleaved by a protease during protein purification. Genes encoding C-Pro domains for the other human fibrillar collagens were purchased from Genewiz and cloned into the same pcDNA3.1 vectors downstream of a preprotrypsin signal sequence and either an HA or FLAG epitope tag. The C-Pro gene for *Col5A2* was inserted using *Not*I and *Xba*I sites. All other collagen C-Pro-encoding genes were inserted between the *Not*I and *Eco*RV sites. FLAG-tagged type-II, type-III, type-V, type-XI, type-XXIV, and type-XXVII genes were further modified by site-directed mutagenesis to introduce the indicated Ser-encoding or Cys-encoding mutations using the Agilent QuikChange II XL Kit and following the manufacturer’s instructions (see Supplementary Table 3 for oligonucleotide sequences). See Supplementary Note 1 for relevant cDNA sequences.

### Cell culture

Adherent HEK293 cells (ATCC) were cultured in Dulbecco's modified Eagle's medium (DMEM) supplemented with l-glutamine, penicillin/streptomycin, and 10% fetal bovine serum. HEK293 Freestyle suspension cells (Thermo Fisher Scientific) were cultured in Freestyle media. Cells were periodically tested for mycoplasma contamination using the Agilent MycoSensor PCR Assay Kit and were negative. HEK293 Freestyle cells were used only in experiments where large quantities of recombinant protein were produced. Transient transfections of pcDNA3.1 C-Pro domain-encoding plasmids were performed using Lipofectamine 3000 (Thermo Fisher Scientific). For all experiments not involving protein purification, transfection media were changed to fresh DMEM 24 h post transfection. Media and lysates were harvested from cell culture 24–72 h post transfection for analysis. When cell lysate samples were required, cells were harvested and then lysed on ice for 10 min in lysis buffer containing 50 mM Tris-HCl, pH 7.5, 150 mM sodium chloride, 1 mM EDTA, 1.5 mM magnesium chloride, 1% Triton X-100, and protease inhibitor tablet (Thermo Fisher Scientific).

### Immunoprecipitations

Adherent HEK293 cells (7.5 × 10^6^) were transfected with 1 μg of DNA encoding the indicated proteins using Lipofectamine 3000 according to the manufacturer’s protocol (Thermo Fisher Scientific). The media were changed 24 h post transfection. Conditioned media were collected 72 h post- transfection, and immunoprecipitated with either HA-antibody agarose beads (Sigma) or FLAG-antibody agarose beads (Sigma). During the immunoprecipitation, samples were mixed end-over-end at 4 °C overnight. The following day, each sample was centrifuged at 2000 × *g* for 5 min at 4 °C, the supernatant was removed, and the agarose beads were washed three times with 50 mM Tris-HCl at pH 7.5 containing 150 mM sodium chloride, 1.5 mM magnesium chloride, 1% Triton X-100, and 50 mM EDTA to disrupt non-covalent Ca^2+^-based assembly products. After the third wash, each sample was eluted using 100 μg/mL FLAG peptide in 1× phosphate-buffered saline (PBS) and mixed end-over-end at 4 °C for 30–60 min. The elution was collected and diluted to 1 mL in 1× PBS to perform the second immunoprecipitation (as indicated), following the same protocol. Elution of the second immunoprecipitation was performed by boiling the beads in 6% SDS and 300 mM Tris at pH 6.8 for 10 min.

### Immunoblotting

Prior to SDS-PAGE analyses, media samples were treated with 100 mM iodoacetamide (Acros Organics) in the dark for 1–2 h to prevent disulfide shuffling, or 100 mM dithiothreitol for 1 h at room temperature to reduce disulfides. All samples were then treated with 6× gel loading buffer (300 mM Tris, pH 6.8, 15% glycerol, 6% SDS, and 10% (w/v) bromophenol blue) and boiled for 10 min prior to protein gel electrophoresis. Samples were then separated by SDS-PAGE using 12% polyacrylamide gels and analyzed by immunoblotting using a LiCor  Odyssey imager for detection. Nitrocellulose blots were probed with primary antibodies (diluted in 5% bovine serum albumin) obtained from the following suppliers: Santa Cruz: HA probe (1:200; sc-7392); Agilent Technologies: rat Anti-DYKDDDDK (1:2000; 200474); and Sigma: β-actin (1:5000; A1978). Secondary antibodies were obtained from LiCor Biosciences: 800CW goat anti-mouse, 800CW goat anti-rat, 680LT goat anti-mouse, and 680LT goat anti-rat. All secondary antibodies were used at a dilution of 1:10,000 in 5% non-fat milk. Uncropped scans of selected immunoblots are supplied in Supplementary Fig. 7.

### Collagen C-Pro expression and purification

HEK293 Freestyle cells (Thermo Fisher Scientific) were transfected at a cell density of 1.0 × 10^6^ cells/mL in 80 mL of Freestyle media with 53 μg of appropriate plasmids using 293Fectin (Life Technologies) at a 2:1 ratio of 293Fectin:DNA. Cells were split 1:2 3 days post-transfection and the supernatant was harvested 6 days post-transfection after pelleting the cells by centrifugation. Clarified media was supplemented with 50 mM potassium phosphate at pH 7, 5 mM imidazole, 150 mM sodium chloride (±0.5 mM CaCl_2_), and 10 mM Tris (final concentrations). The protein was bound to Ni-NTA resin by gravity flow, washed with 300 mM sodium chloride, 10 mM imidazole (±0.5 mM CaCl_2_), and 150 mM potassium phosphate at pH 7, and then eluted with 300 mM sodium chloride, 250 mM imidazole (±0.5 mM CaCl_2_), 150 mM potassium phosphate at pH 7, into either a mixture of CaCl_2_ and EDTA (to chelate nickel ions) at a final concentration of 2 mM CaCl_2_ and 1 mM EDTA, or solely 30 mM EDTA. The eluted proteins were then concentrated to a final volume of 1–5 mL using a 10 kDa molecular weight cutoff (MWCO) filter (protein concentration was not measured at this stage), buffer exchanged into 20 mM Bis-Tris propane pH 7, 150 mM sodium chloride (±0.5 mM CaCl_2_), and subjected to overnight cleavage with 6× His-tagged HRV-3C protease (Pierce). The cleaved protein mixture was applied to a new Ni-NTA column, such that the cleaved His epitope and the His-protease bound to the beads while the tag-less, purified protein was collected. The sample was then concentrated to 250 μL total volume (protein concentration was not measured at this stage) and further purified by a Superdex 200 10/30 GL column. Purified protein samples were collected and quantified post-size exclusion chromatography (SEC) analysis, with all proteins being concentrated down to <1 mL, attaining final concentrations ranging from 0.2 to 1 mg/mL. Coomassie gels showing the purified protein samples are presented in Supplementary Fig. 2.

### Analytical ultracentrifugation

Sedimentation equilibrium experiments were performed with a Beckman XL-A analytical ultracentrifuge to evaluate the assembly of wild-type C-Proα1(I), wild-type C-Proα2(I), C2S C-Proα1(I), and S2C C-Proα2(I). Data were collected at 25 °C with gradients monitored at 275 nm in a Beckman XL-A analytical ultracentrifuge. Pathlength double-sector, charcoal-filled Epon centerpieces (1.2 cm) were used for all samples with ~100 μL on the sample side and ~110 μL of buffer or water as the reference. In most instances, three samples of a construct were spun, and diluted as required. Equilibrium data used in the analyses were collected at four or more speeds for each sample with equilibrium ascertained as superimposable gradients collected ≥2 h apart. A non-sedimenting contribution was measured after high-speed depletion of protein. This component was generally minor (<0.04 a.u.) and treated as a fixed, measured parameter in the final data analyses. In some instances, after attaining equilibrium at the highest speed, the speed was reduced to one of the earlier speeds and the sample allowed to re-equilibrate. In all tested cases, the gradients were nearly identical, suggesting no irreversible loss of material during the experiment.

For the variants, the sequence molecular weights (*M*_S_) and the partial specific volumes ($\bar v$) were computed from the sequences using tabulated values^39,40^. While the polypeptides were shown to be glycosylated, the extent and specific nature of the glycosylation was unknown and likely heterogeneous. However, matrix assisted laser desorption ionization-time of flight mass spectrometry (MALDI-TOF) mass spectra yielded masses (shown in Supplementary Table 4) only slightly higher than the sequence weights. Therefore, the potential impact of glycosylation on $\bar v$ values was ignored. The buffer used for sedimentation equilibrium experiments was 20 mM Bis-Tris propane at pH 7 with 150 mM NaCl, with or without 0.5 mM CaCl_2_. Buffer density was measured at 25 °C by an Anton Paar DMA 5000 to be 1.005 g/mL, and so in the analysis a value of 1.0 was used. The extinction coefficients at 280 nm were calculated based on average values for *W* and *Y*, ignoring potential contributions from disulfides^41^. In equilibrium constant calculations, this extinction coefficient was used without adjustment for the 275 nm wavelength used in data collection. These molecular properties are summarized in Supplementary Table 4.

Data were analyzed using an approach similar to that described by Laue^27^, in which various models containing one or two species were fit to all the data collected for a given variant. The analysis was implemented in software written for Igor Pro (WaveMetrics Inc., Portland, OR, USA) by Darrell R. McCaslin of the Biophysics Instrumentation Facility at the University of Wisconsin–Madison. To minimize the impact of errors in various molecular parameters and solvent properties on the fits, each species was treated as a reduced molecular weight $M_{\mathrm{R}} = M_{\mathrm{P}}(1 - \bar v\rho )$, where *M*_P_ is the weight-average molecular weight, which for a single species would be the polypeptide weight, or a multiple of it if a single oligomer. A single species model has one such term, a two-species model has two such fitting terms which may or may not have an a priori relationship between the species (e.g., one species is a dimer of the other); the two-species model can be elaborated to include an equilibrium among species. The slope of a plot of the logarithm of the absorbance as a function of the squared distance from the center of rotation (*r*^2^) is proportional to *M*_R_ and other measurement constants; however, when data are plotted as $\frac{{2RT}}{{\omega ^2}}{\mathrm{ln}}(A)$ versus *r*^2^, the slope is *M*_R_ and is directly related to the molecular weight (as shown in Fig. 4b–e).

Both wild-type C-Proα1(I) and S2C C-Proα2(I) in the absence of Ca^2+^ were best fit as a single species with no direct evidence for a second species present in solution. From the reduced molecular weights and using the $\bar v$ values from Supplementary Table 4, the molecular weights are 88,500 and 90,900 Da, respectively. These values are most consistent with the species being homotrimers, with molecular weights approximately 2000 Da larger than the sequence mass, consistent with some degree of glycosylation. All the C-Pro constructs employed had a single N-glycosylation site in the polypeptide. Examples of fits to the observed gradients are shown in Supplementary Figs. 3a and b. In the presence of 0.5 mM Ca^2+^, both wild-type C-Proα1(I) and S2C C-Proα2(I) were again best fit as trimers and example fits to the observed gradients are shown in Supplementary Fig. 3c, d.

For C2S C-Proα1(I) and wild-type C-Proα2(I) in the absence of Ca^2+^, over the range of concentrations employed both protein samples could be fit as a single species, but with molecular weights intermediate between a monomer and dimer of the respective polypeptide chains ($\frac{{M_{\mathrm{W}}}}{{M_{\mathrm{S}}}}$=1.597 and 1.38, respectively). As the MALDI-TOF data do not show evidence of such a high molecular weight, the data are best viewed as an equilibrium between monomer and dimer. For C2S C-Proα1(I), an equilibrium constant of 76,300 M^–1^ provided the best fit, while for wild-type C-Proα2(I) an equilibrium between a species with $\frac{{M_{\mathrm{W}}}}{{M_{\mathrm{S}}}}$=1.17 and an equilibrium constant of 6500 M^–1^ provided the best fit. The small magnitudes of these equilibria constants are consistent with the mixtures approximating a single species of intermediate molecular weight over the full concentration range employed. Examples of these fits are shown in Supplementary Fig. 4a for wild-type C-Proα2(I) and in Supplementary Fig. 5a for C2S C-Proα1(I) with the relative contributions of the monomeric and dimeric species to the overall gradients also computed and shown, highlighting that the monomer was the predominant species in both cases.

For the same variants (C2S C-Proα1(I) and wild-type C-Proα2(I)) in the presence of 0.5 mM Ca^2+^, the presence of homotrimeric species was apparent. Shown in Supplementary Fig. 4b for wild-type C-Proα2(I) and in Supplementary Fig. 5b for C2S C-Proα1(I) are examples of best fits to the observed gradients modeling the data as monomer–trimer equilibria with the relative contributions of the monomeric and trimeric species to the overall gradients also computed and shown, highlighting that the trimer was the predominant species in both cases.

### Disulfide mapping by mass spectrometry

Wild-type C-Proα1(I) obtained from SEC purification was treated with 55 mM iodoacetamide for 1 h in the dark to covalently modify any free cysteine residues and minimize the potential for disulfide shuffling. The *N*-glycan was removed by denaturing the protein by boiling for 10 min in 0.5% SDS, followed by incubation with PNGase-F (NEB) for 2 h at 37 °C. PNGase-F was removed by centrifugation over a 50 kDa MWCO filter. The protein was precipitated with trichloroacetic acid, and the resultant pellet was washed with acetone. The pellet was resuspended in 8 M urea, and then diluted to 0.38 M urea in 100 mM ammonium acetate at pH 4.0. The protein was digested with endoproteinase GluC (Pierce) at a ratio of 1:20 substrate:enzyme overnight at 37 °C. Proteolyzed samples were quenched by the addition of formic acid to a final concentration of 5% and applied to Protea C18 SpinTips. The eluent from the SpinTips was evaporated completely by speed vacuum centrifugation, and the peptides were resuspended in 25 mM Tris and 1 mM EDTA at pH 7.0. Peptides were digested again with rLys-C (Promega) at a ratio of 1:27 substrate:enzyme overnight at 37 °C.

S2C C-Proα2(I) was treated in the same manner as wild-type C-Proα1(I) through the trichloroacetic acid precipitation and acetone wash. The pellet was resuspended in 8 M urea, and then diluted to 0.38 M urea in 100 mM ammonium bicarbonate at pH 6.4 for trypsin digestions or in PBS at pH 6.8 for endoproteinase GluC digestions. The pH of all samples was maintained below 7 to minimize disulfide shuffling. The protein was digested with trypsin (Pierce) at a ratio of 1:40 substrate:enzyme overnight at 37 °C or with endoproteinase GluC at a ratio of 1:20 substrate:enzyme for 2 days at 37 °C.

All proteolyzed samples were quenched by the addition of formic acid to a final concentration of 5% and desalted using Protea C18 SpinTips. The eluent from the SpinTips was evaporated by speed vacuum centrifugation, and the remaining peptides were resuspended in 0.1% formic acid. Samples were injected onto an EASY-nLC 1000 with mass spectrometry (MS) data acquired on a Thermo Q Exactive mass spectrometer. MS spectra were manually searched for the predicted masses of each set of disulfide-linked peptides. For each digestion condition, all peptide masses listed were observed in the same sample data set. Additionally, all charge states listed for each peptide were observed in the same MS spectra.

## Electronic supplementary material

Supplementary Information

## Data Availability

All data generated during and/or analyzed during the current study are either included in this published article and its Supplementary Information file, or are available from the corresponding author on reasonable request.
