# Supplementary material for: A cysteine-based molecular code informs collagen C-propeptide assembly
Source: Nat Commun. 2018 Oct 11;9:4206. doi: 10.1038/s41467-018-06185-2 (PMC6181919; doi:10.1038/s41467-018-06185-2)
Supplement: Supplementary file 1 — Supplementary Information [file 41467_2018_6185_MOESM1_ESM.pdf]

A Cysteine-Based Molecular Code Informs Collagen C-Propeptide Assembly

DiChiara, A.S. and co-workers

Page	Contents
S1	Table of Contents
S2–S10	Supplementary Figures
S11–12	Supplementary Tables
S13–16	Supplementary Note

SUPPLEMENTARY FIGURES

```
                                                                           C1    C2
sponge_ColF1         ------------------VNLGSVADVIELHKKLQHL--KSPTGT-KDSPARSCHDLFLE      382
Hydra_colα1(I)       ---------------EKNKDTVI-EKLIRLEDITQGA--QKPDGS-EYFPAKSCKDLKMC      1262
Hydra_col1           ----------------TEIENNFNNRVKILKSSVEAY--KKPNGS-KEFPARTCRDIYAF      1213
Hydra_col2           ---------------EKNKDTVI-EKLIRLEDVTQGA--QKPDGS-EYFPAKSCKDLKMC      1286
Hydra_col3           ------LVKLIKR-----TQENLRNFNKVWNTVLDDLVIKNELGT-RMHPAITCRHVFVN      1690
Hydra_col5           ------VDEIIKR-----NIDGFDTMYRVFKT-ADKV-FLGGDGT-RDNPARSCLELFQW      1194
Red fire ant_α1(I)   ---------------------------ENLKSSFQKL--VKPDG-EKNSPAKTCRDLFV-      29
Mollusk_α1(I)        -------------------QYE---NLNRVREAIVRI--GGTRLGSRTSPGKNCRDIKLS      734
Sea urchin_Col1Pa    ----------------KIQDTELLGAISALGQQIELI--KAPQGKAKTNPARSCKDVFLN      3362
Sea urchin_Col2Pa    ------------------DRTQFQIYLAKFESEILSL--IEPLGS-RDQPIRSCKDLFKC      3008
lancelet_α1(I)       ----------------IGKDTKVSDALEAISAQIESL--KKPTGT-RKNPARTCVDLALC      217
lancelet_α2(I)       STDIDHLKKLISRVGPAEWTDYFEGEMDRLQGIVKTI-VGGPWGT-AEYPAKTCKDLMFA      2093
Tunicate_α1(I)       ----------------PEGLEEIYAAMETLKQELEMM--KEPMGRTQDNPGRSCKDIWLC      1177
Tunicate_α2(I)       -------QPVTSD---VGRDPEMMLVLKELTSSVEDI--KAPRGVSRKTPARSCLDIYLA      1225
Lamprey_α1(I)        ----------------LPYGLVLDASIKALNAQVESI--TSPDGS-RKHPARSCRDLMLC      1078
Lamprey_α2(I)        ----------------GERNLELEASISSLNVRVENM--VSPDGS-QKNPARTCRNLKLC      992
Shark_α1(I)          ----------------RNRDLEVDTTLKSLSQQIENI--RSPEGT-KKNPARTCRDLKMC      1256
Shark_α2(I)          ----------------SAKDFEVDATLKQLSSQIETL--LFPEGS-KKNPARTCRDLRLS      1168
Coelacanth_α1(I)     ----------------RDRHAEVETTLKSLTKQIDNI--RSPEGT-RKNPARTCRDLKMC      1262
Coelacanth_α2(I)     ----------------TEKDLEVDSTLKVLTNQIESI--RTPEGS-RKNPARSCRDLRLS      1169
Frog_α1(I)           ----------------RDRDHEVDSTLKSLSKQIENI--QSPEGT-RKNPARTCRDLKMC      1250
Frog_α2(I)           ----------------RPKDYEVDATLKSLNNQIETI--LTPEGS-KKNPARTCRDLRLS      1156
Human_α1(I)          ----------------RDRDLEVDTTLKSLSQQIENI--RSPEGS-RKNPARTCRDLKMC      1265
Human_α2(I)          ----------------RPKDYEVDATLKSLNNQIETL--LTPEGS-RKNPARTCRDLRLS      1169
                                                                      ••
                                            C3       C4             C5
sponge_ColF1         --DNST--SD--GYYWIDPNGGCIGDAVKVFCNFTGG-----VQQTCISATKNAG-----      426
Hydra_colα1(I)       --HPNV--IS--GEYYIDPSLGANVDKIKVTCEFY-----PTASETCIKPTVSMF-----      1306
Hydra_col1           --YPDS--SS--GMYYIDPNKGCIDDAIYVHCNFTKSHDEETKITTCVYPEKTMS-----      1262
Hydra_col2           --HPNV--IS--GEYYIDPSLGANVDKIKVTCEFY-----PTASETCIKPTVSMF-----      1330
Hydra_col3           --NNNS--KS--GDYWIDPNEGSPVDAFLVYCNAS-------TLETCIFPKQPLV-----      1732
Hydra_col5           --HLGA--NT--GDYWIDPNEGSPDDSVLVHCNKL-------TNETCVYSKNTQI-----      1236
Red fire ant_α1(I)   A-YPD----KLSGEYWIDPNEGDIRDAILVYC---DAE--KRA--TCILPNP--S--RSP      73
Mollusk_α1(I)        --NPDF--KD--GDYWIDPNGDSALDAVKVFCRM-------ETLETCVKPKFIEY-----      776
Sea urchin_Col1Pa    --NVEA--ES--GYYWVDPNLGCQKDAIQVYCEA-------ETGATCVPSTNNVV-----      3404
Sea urchin_Col2Pa    --YPEA--ED--GNYWIDSNEGSVKDAFLAHCVKRGE---SGSPETCITPRVDEI-----      3054
lancelet_α1(I)       --HPTW--PS--GNYWIDPNQGCTVDAIEVWCDM-------KTLETCVYPKPAKV-----      259
lancelet_α2(I)       --QPGL--KD--GYYFIDPDGGCIENAFQAFCNFT------AGGLTCFQPTNDTL-----      2136
Tunicate_α1(I)       --HPDF--PS--GNYWIDPNGGCSADAIEVFCDFE------AEGDTCISPVERTASVSWL      1225
Tunicate_α2(I)       --EQQQGTVPKSGVRWIDPNGGCNADGLEVYCNF-------HTMETCVYPTNRNI-----      1271
Lamprey_α1(I)        --HPEY--KSDTGEYWIDPNEGCRADAIKVWCNM-------ETGESCVNPGMPSL-----      1122
Lamprey_α2(I)        --YPDL--PS--GLYWIDPNEGSKSDAIQVWCNM-------ETGESCMNADNPSI-----      1034
Shark_α1(I)          --HPEW--KS--GDYWIDPNQGCSLDAIRVYCNL-------ETGETCVYSSPQSI-----      1304
Shark_α2(I)          --HPEW--KS--GYYWIDPNQGCTQDAIRVFCEF-------TTGETCVHASPDII-----      1211
Coelacanth_α1(I)     --HPEW--KS--GDYWIDPNQGCMLDAIKVYCNM-------ETGETCVYPTQSSV-----      1298
Coelacanth_α2(I)     --HPEW--SS--GFYWIDPNQGCTMDAIKVYCDF-------TNGETCINANPETI-----      1210
Frog_α1(I)           --HSDW--KS--GEYWIDPNQGCILDAIKVYCNM-------ETGETCVYPTQNSI-----      1292
Frog_α2(I)           --HPDW--SS--GFYWIDPNQGCTSDSIRVFCDF-------STGETCIHANPDSI-----      1198
Human_α1(I)          --HSDW--KS--GEYWIDPNQGCNLDAIKVFCNM-------ETGETCVYPTQPSV-----      1307
Human_α2(I)          --HPEW--SS--GYYWIDPNQGCTMDAIKVYCDF-------STGETCIRAQPENI-----      1211
                                      • •• •  •

sponge_ColF1         DLKS--WSGH-------SIWFSDM-LGGFKLTYDIS------------------------      452
Hydra_colα1(I)       EKKK--WVSQST---DQWKWFNGEISSDSIFTYASQ------------------------      1337
Hydra_col1           VEKD--SWPTKL-HTKAQRWFVEDH-ELGKLSYAAD------------------------      1294
Hydra_col2           EKKK--WVSQST---DQWKWFNGEISSDSIFTYASQ------------------------      1361
Hydra_col3           EKAD--WFTGK----DHLMWAYKDILAEGGITYSSD------------------------      1762
Hydra_col5           SKNN--YFKDNT---DSYKWLMTEVA--NNFDYAME------------------------      1265
Red fire ant_α1(I)   NITH-ITE-----QQET--WLSE-IDNG----MK-----------------------I-T      96
Mollusk_α1(I)        RRDR--WTKDTT---SGQYFMYDVFGKVKQFKYDID------------------------      807
Sea urchin_Col1Pa    SNMT--WYVGKT----KR-AFFSSMHGGDKFAYIED------------------------      3433
Sea urchin_Col2Pa    SRAR--WYEGASG----SRYI--TEMGLEKFSYEAS------------------------      3082
lancelet_α1(I)       PKAS--WYNGP----AKHVWFSESIKGGYQFGYTAD------------------------      289
lancelet_α2(I)       ASPR--SPYK----VDNFTWFSEV-EGGFEIEYEGD------------------------      2165
Tunicate_α1(I)       TSKR--WPKA-----QPGDWFSSY-RMGDRFEYNTS------------------------      1253
Tunicate_α2(I)       ENGT--HYTGE----PGHTYYGEEMTRVEHADY---------------------------      1298
Lamprey_α1(I)        PRKN--WWRSQA-ADKKHVWLGETMSPGSQFTYGDG------------------------      1155
Lamprey_α2(I)        ARKN--WWLKPS-GSKKHVWFGVTMSPDAQFTYGED------------------------      1067
Shark_α1(I)          PQKN--WYTSKNPKEKKHIWFGESMNGGFQFGYGADD-----------------------      1339
Shark_α2(I)          ERRN--WWISNDAQEKKHIWFGETIKDGAQFTYNEE------------------------      1245
Coelacanth_α1(I)     PQKN--WYVSKNPKDKKHIWFGETMNEGFQFEYGSE------------------------      1332
Coelacanth_α2(I)     PSKT--WYISKNPMDKKHIWFGETINGGTQFEYNDE------------------------      1244
Frog_α1(I)           AQKN--WYTSKNPREKKHVWFGEAMSDGFQFEYGSE------------------------      1326
Frog_α2(I)           PQKN--WYTSKT-KQRKHVWFGETINGGTQIEYNEE------------------------      1231
Human_α1(I)          AQKN--WYISKNPKDKRHVWFGESMTDGFQFEYGGQ------------------------      1341
Human_α2(I)          PAKN--WYRSS--KDKKHVWLGETINAGSQFEYNVE------------------------      1243
```

```
                                                                  C6
sponge_ColF1          ----------------------------RSQLQFIRAASRHAVQSFTYKCRNSAAAVIFR       484
Hydra_colα1(I)        -----------------------------VQLRFLRLNSQFVRQNLTYHCLNSHAHNDSF       1368
Hydra_col1            ----------------------------QSQLTFLGYLSREAYQNVTFHCQNTLVWYDKQ       1326
Hydra_col2            -----------------------------VQLRFLRLNSQFVRQNLTYHCLNSHAHNDSF       1392
Hydra_col3            ----------------------------MVQLKMMQLLSAKCRQNITYFCKNSYSNITIK       1794
Hydra_col5            ----------------------------TPQMKILSLLSTTVRQTITFHCKNIPLLRGA-       1296
Red fire ant_α1(I)    YK----A-D--------------SNQISF-----LQLLSKHANQNITYHCKNSVAYFDY-       131
Mollusk_α1(I)         ----------------------------AYQLKVLQFDSQAARQGVIYHCLNSHTYGTRF       839
Sea urchin_Col1Pa     ----------------------------STQMTFLRLLSTSARQTVTYFCKNVQ------       3459
Sea urchin_Col2Pa     ----------------------------EVQLTFLRLLSTKAHQNVTYHCKNSVAVRDRQ       3114
lancelet_α1(I)        ----------------------------DIQMQFLRLSSTSARQNVTYHCKNSVAYYDAV       321
lancelet_α2(I)        ----------------------------PIQLNYIQALSTRATQTFTFECSSAVAWYNWN       2197
Tunicate_α1(I)        ----------------------------IPQFNFLRLLSSQAKQRFTYKCVNSIGWENQQ       1285
Tunicate_α2(I)        ----------------------------ASQLTFLRLLSSKAKQQVTFFCRNMVAYYDAS       1330
Lamprey_α1(I)         ------------------------SPNMEVQLTFLRLLSTDASQKITYHCKNSVAYLDSR       1191
Lamprey_α2(I)         ------------------------SHSTEIQLTFLRLFSTEASQKITYHCKNSVAYQDGA       1103
Shark_α1(I)           ---------------------GVLASDVAIQMTFLRLMSSEATQNVTYHCKNSIAYMDEE       1378
Shark_α2(I)           ---------------------HITPLVMATQLTFLQLLSNEASQNVTYYCKNSIAYMDEE       1284
Coelacanth_α1(I)      ---------------------GSDPADVAIQLTFLRLMSTEASQNITYHCKNSIAYMDQQ       1371
Coelacanth_α2(I)      ---------------------SITPKIMATQLAFMRLLANQASQNITYHCKNSIAYMDEQ       1283
Frog_α1(I)            ---------------------GSDPADVAIQLTFLRLMATEASQNITYHCKNSVAYMDQA       1365
Frog_α2(I)            ---------------------GVTSKDMATQFAFMRLLANHASQNITYHCKNSIAYMDGQ       1270
Human_α1(I)           ---------------------GSDPADVAIQLTFLRLMSTEASQNITYHCKNSVAYMDQQ       1380
Human_α2(I)           ---------------------GVTSKEMATQLAFMRLLANYASQNITYHCKNSIAYMDEE       1282
                                                                C7
sponge_ColF1          TQDN----------------KEIA------------ANKVTYDGC----------KSRPS       506
Hydra_colα1(I)        GNNRPYVKI------MSSDDIEIH---TGSHMKNRL--KVLQDQC----------NKKDN       1407
Hydra_col1            NNDY-----KKAMKFKGTEDQEFA---YSEDKQSMFMPHAVNDEC----------SNMSK       1368
Hydra_col2            GNNRPYVKI------MSSDDIEIH---TGSHMKNRL--KVLQDQC----------NKKDN       1431
Hydra_col3            TDENVHLHIHNGKISINPDETYIR---HGIALKLNL--MAIKDDC----------KVKDD       1839
Hydra_col5            K-NTIKFKLDNG---------AIY---HKKMRGVVL--KVITDDC----------TV-EN       1330
Red fire ant_α1(I)    ----------GNPIFLSSSDVELV---DDQDSKFNY--RTLEDGC----------SSSSS       3494
Mollusk_α1(I)         TGST-----EQALRLMTTSDVELS---LDAPSQEQY--EVIEDGC----------QERSA       3154
Sea urchin_Col1Pa     -E-RKTY--RRSLKLLAWNDAE-LSPR-GN-QRLRY-EMI-MDECR--LHQ--------N       172
Sea urchin_Col2Pa     IT-------------DSGDELDSA---EGRFKRSTYI-DILEGECEN-------VSSKDN       875
lancelet_α1(I)        SSNY-----KKGLMLMGNGETELG---AQGPKKYQL--YAVEDGC----------QTPTG       361
lancelet_α2(I)        TDGY-----DQAVRLLSNNENVLT---YGTPGV-----KTIYDGC----------QFASP       2234
Tunicate_α1(I)        TGSF-----DQAIHLLAANDEVLT---YGSEHL-----TVIEDNC----------KT-GH       1321
Tunicate_α2(I)        ADNK-----AQALKLRGFGDAEFT---AEGAVGTTY--RVLHDGC----------STRPT       1370
Lamprey_α1(I)         AGNL-----KKALMLQGSSDVEIR---AEGNSRFTY--SVLEDGC----------TTHTG       1231
Lamprey_α2(I)         VGNL-----QQALLLQGSNEMEIR---AEGNSKLAY--SVLEDGC----------TMHTG       1143
Shark_α1(I)           AGNL-----KKAVLLQGSNEIEIR---AEGNSRFTY--SVSEDGC----------TRHTG       1418
Shark_α2(I)           SGSL-----KKAVLLLGNSDVELR---AEGNSRFTY--SVLEDGC----------TKHTG       1324
Coelacanth_α1(I)      AGNL-----KKSLLLQGSNEIEIR---AEGNSRFTY--SVTEDGC----------TRHTG       1411
Coelacanth_α2(I)      TGNL-----KKAVMLQGSNDVELR---AEGNSRFTF--SVLEDGC----------TKHTS       1323
Frog_α1(I)            TGNL-----KKALLLQGSNEIEIR---AEGNSRFTY--SVVEDGC----------TQHTG       1405
Frog_α2(I)            TGNL-----KKAILLQGSNDVELR---AEGNSRFTF--SVLEDGC----------TQHTG       1310
Human_α1(I)           TGNL-----KKALLLQGSNEIEIR---AEGNSRFTY--SVTVDGC----------TSHTG       1420
Human_α2(I)           TGNL-----KKAVILQGSNDVELV---AEGNSRFTY--TVLVDGC----------SKKTN       1322
                                                             C8
sponge_ColF1          VPDAAFVAVETKRVEQLPIRDFASSDIAGQHQEFGFEMGPACFY-        550
Hydra_colα1(I)        QWHKTVFEFSSKITSRLPIVDVAVFDVANVGEQFGIELGPVCFY-        1451
Hydra_col1            EWRTTTLRFTSRKYIRLPIIDFAPISSEDNNAMFGVELGPVCFM-        1412
Hydra_col2            QWHKTVFEFSSKITSRLPIVDVAVFDVANVGEQFGIELGPVCFY-        1475
Hydra_col3            KWRESVFEITSNRLENLPIQDIGVTDIADDGEQFGLLIGPVCFS-        1883
Hydra_col5            KWQKLVLELSTSKNEILPVIDLGVHGNGITSSEIGVDIGPACFS-        1374
Red fire ant_α1(I)    QWGKSVYEYETKKTTRLPIVDFAPGEVGSESQMFGLEMGPVCFS-        3538
Mollusk_α1(I)         EWSQTVINYSTRRNTRLPIVDVAPSDIGGEGQEFGITLGPVCFS-        3198
Sea urchin_Col1Pa     HWGKTVVSYETDKPVRLPIIDVALRDIGNPDQSFSIEIGAACY--        215
Sea urchin_Col2Pa     QWHTNRYEVRTNKSELLPLVDVLLFDIGGENQQFGIDVGEVCFS-        919
lancelet_α1(I)        KWSSTVLEYKTKKTTRLPFTDIAPYD-------------------        387
lancelet_α2(I)        QLDLTVIEINTTATECVPVRDFGVFELDENGQEFGFSVGQVCFQ-        2278
Tunicate_α1(I)        GNGQVVLELRTREVDLLPLFDYKAFDFGTRSQRHGYQLDRVCFSG        1366
Tunicate_α2(I)        QWDRTEIEFETRLVGRMPITDIAPFDIGDADQQFGAKFGPVCFK-        1414
Lamprey_α1(I)         VWGKTVIEYRTQKTSRLPFMDIAPMDVGGSDQEFGVDVGPVCFL-        1275
Lamprey_α2(I)         QWGKTVIEYRTPKTSRLPIVDIAPKDVGGPDQEFGVDVGPVCFL-        1187
Shark_α1(I)           EWGRTVIEYKTMKTSRLPIIDIAPMDVGGADQEFGVEIGPVCFL-        1462
Shark_α2(I)           EWSKTIIEYRTQKTSRLPFVDIAPMDIGGPEQEFGLDIGPVCFK-        1368
Coelacanth_α1(I)      AWGRTVIDYKTTKTSRLPVIDIAPMDVGAPDQEFGIDIGPVCFL-        1455
Coelacanth_α2(I)      QWGKTVIEYRTNKPTRLPILDIAPMDIGGADQEFGLDIGPVCFK-        1367
Frog_α1(I)            EWGKTVIDYKTTKTSRLPITDVAPMDIGAPDQEFGIDIGPVCFV-        1449
Frog_α2(I)            EWGRTVIEYRTNKPSRLPILDIAPLDIGGDDQEFGLDIGPVCFK-        1354
Human_α1(I)           AWGKTVIEYKTTKTSRLPIIDVAPLDVGAPDQEFGFDVGPVCFL-        1464
Human_α2(I)           EWGKTIIEYKTNKPSRLPFLDIAPLDIGGADQEFFVDIGPVCFK-        1366
```

Supplementary Figure 1 | Full alignments of ancestral collagen-I C-Pro domains. Alignment of collagen-I C-Pro domains within the chordate lineage. Cys residues are highlighted in yellow. Amino acids previously proposed to be important for salt bridge formation are bolded in red. Amino acids other than cysteine in the conserved cysteine positions are bolded. The collagen recognition sequence is outlined by a black box in sequences where it is present. Amino acids involved in the Ca^{2+} coordination network are marked with a "•" below the alignment. The accession numbers for each sequence are as follows: sponge_ColF1 P18856, hydra_α1(I) T2MHG7, hydra_col1 AF525468, hydra_col2 A1XVT1, hydra_col3 A1XVT2, hydra_col5 A1XVT3, sea urchin_Col1Pa M92040, sea urchin_ColP2a M92041, red fire ant_α1(I) UniRef90_UPI0005960200, mollusk_α1(I) UniRef90_UPI00084B9720, tunicate_α1(I) H2YGA7, tunicate_α2(I) H2YJN4, lancelet_α1(I) AB193827.1, lancelet_α2(I) XM_019787639.1, lamprey_α1(I) S4R787, lamprey_α2(I) S4RDA0, shark_α1(I) XM_007910713.1, shark_α2(I) XM_007909256.1, coelacanth_α1(I) XM_005992184.2, coelacanth_α2(I) XM_006011624.2, frog_α1(I) NM_001011005.1, frog_α2(I) NM_001079250.1, human_α1(I) NM_000088.3, and human_α2(I) NM_000089.3.

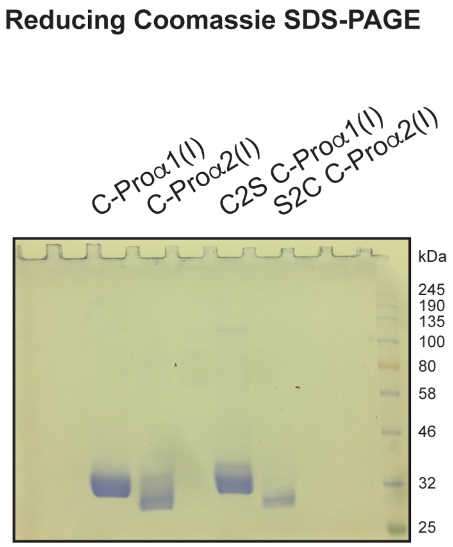

Supplementary Figure 2 | Collagen-I C-Pro purification.

Coomassie staining of a reducing SDS-PAGE gel showing recombinantly prepared, purified samples of wild-type C-Proα1(I), wild-type C-Proα2(I), C2S C-Proα1(I), and S2C C-Proα2(I).

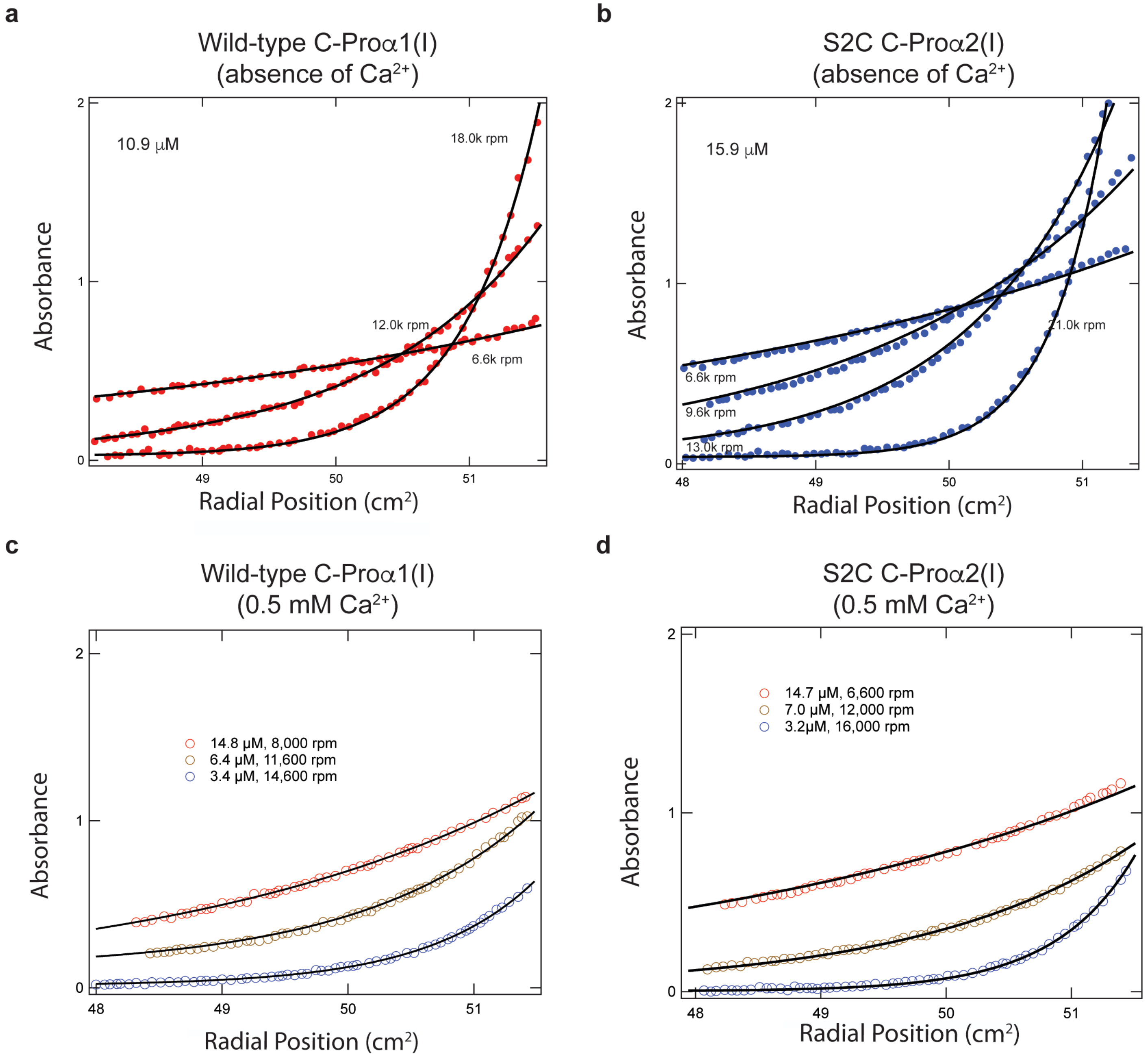

Supplementary Figure 3 | Examples of global fits of the data collected for disulfide-linked homotrimeric C-Pro variants during sedimentation equilibrium analyses in the absence and presence of Ca^{2+}.

(a–b) Single species fits for wild-type C-Proα1(I) and S2C C-Proα2(I) at a single initial concentration and multiple speeds in the absence of Ca^{2+}. The fitted single species molecular weights based on a global fit to all the data available for each variant were 88,500 Da and 90,900 Da, respectively (showing only every second data point for clarity).

(c–d) Examples of global single species fits of the data for wild-type C-Proα1(I) (c) and S2C C-Proα2(I) (d) at several concentrations and speeds (showing only every second data point for clarity) in the presence of Ca^{2+}.

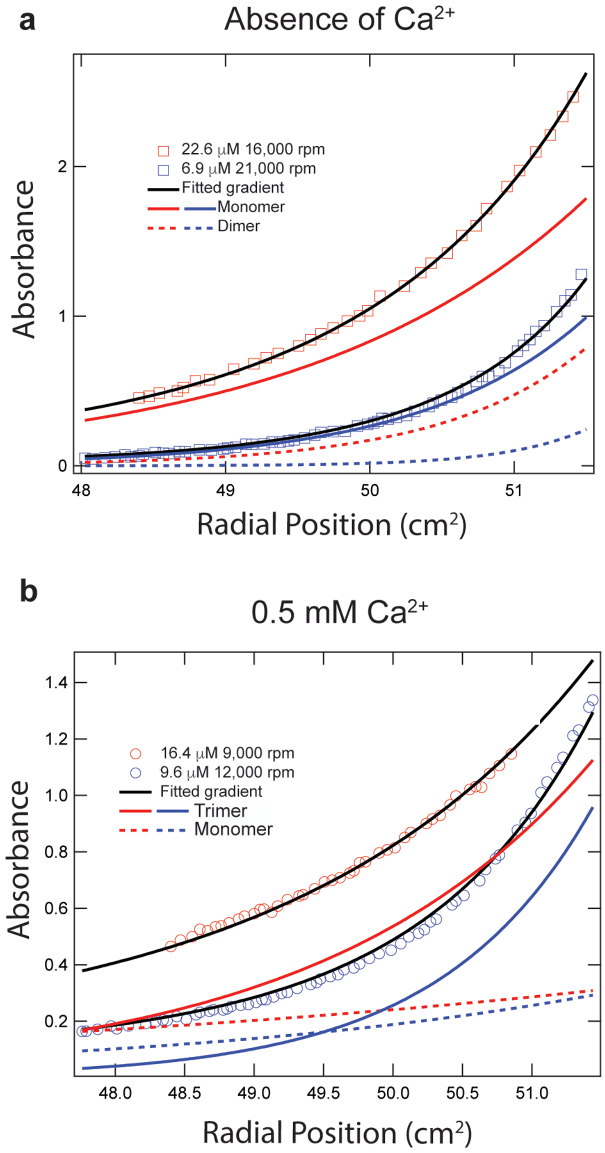

Supplementary Figure 4 | Global fits for wild type C-Proα2(I) in the absence and presence of Ca^{2+}.

(a) Example global fit to a monomer–dimer equilibrium model for wild-type C-Proα2(I) in the absence of Ca^{2+}. The fitted gradient superimposes on the measured data (showing only every second data point for clarity). The contributions of the monomer and dimer species to the total gradient are also shown, based on a fitted equilibrium constant of 6500 M^{-1}. This distribution could be replaced by a single gradient with a molecular weight intermediate to the monomer and dimer, as shown in Fig. 4d.

(b) Example global fit to a monomer–trimer equilibrium model for wild-type C-Proα2(I) in the presence of 0.5 mM Ca^{2+}. The fitted gradient superimposes on the measured data (showing only every second data point for clarity). The contributions of the monomer and trimer species to the total gradient are also shown.

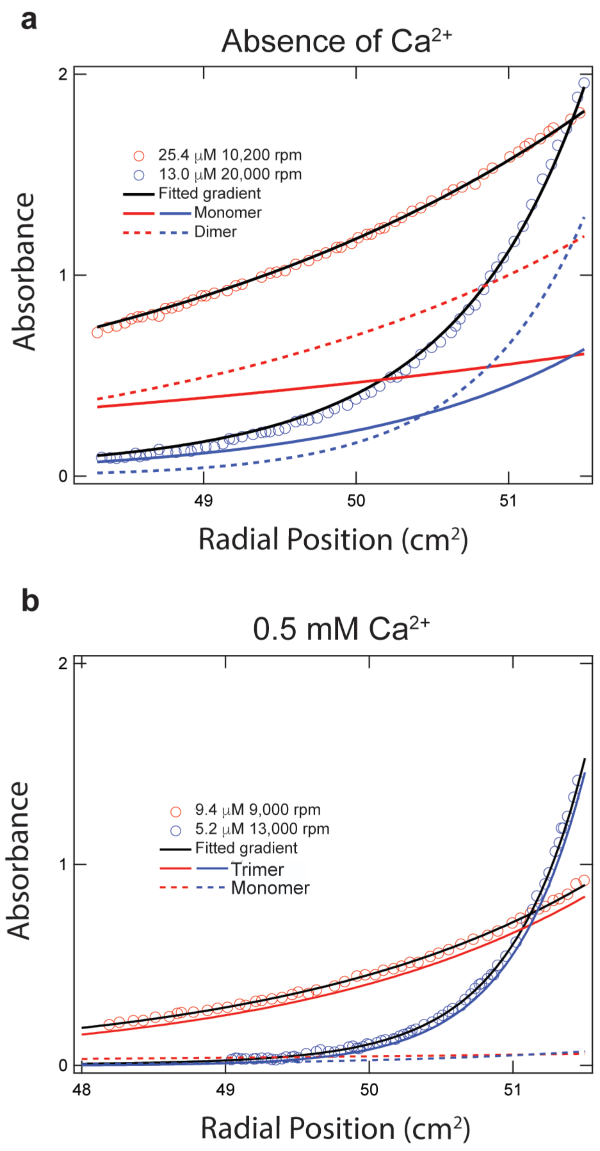

Supplementary Figure 5 | Global fits for C2S C-Proα1(I) in the absence and presence of Ca^{2+}.

(a) Example global fit to a monomer–dimer equilibrium model for C2S C-Proα1(I) in the absence of Ca^{2+}. The fitted gradient superimposes on the measured data (showing only every second data point for clarity). The contributions of the monomer and dimer species to the total gradient are also shown, based on a fitted equilibrium constant of 76,300 M^{-1}. This distribution could be replaced by a single gradient with a molecular weight intermediate to the monomer and dimer, as shown in Fig. 4e.

(b) Example global fit to a monomer–trimer equilibrium model for C2S C-Proα1(I) in the presence of 0.5 mM Ca^{2+}. The fitted gradient superimposes on the measured data (showing only every second data point for clarity). The contributions of the monomer and trimer species to the total gradient are also shown.

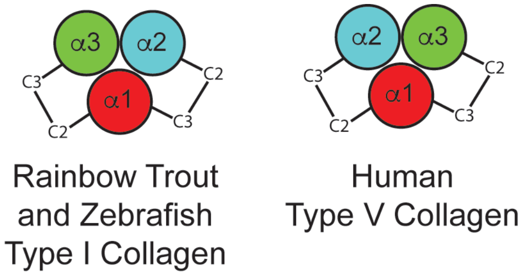

Supplementary Figure 6 | Topology of 1:1:1 heterotrimers.

The cysteine-based code provides insight into the topology of the 1:1:1 heterotrimers that can form. Given the difference in cysteine patterns between Colα2(I) and Colα3(I) of rainbow trout and zebrafish versus Colα2(V) and Colα3(V) in humans, the α2 and α3 chains are likely in different orientations relative to the α1 chain, which in all three species maintains cysteine residues in both the C2 and C3 positions.

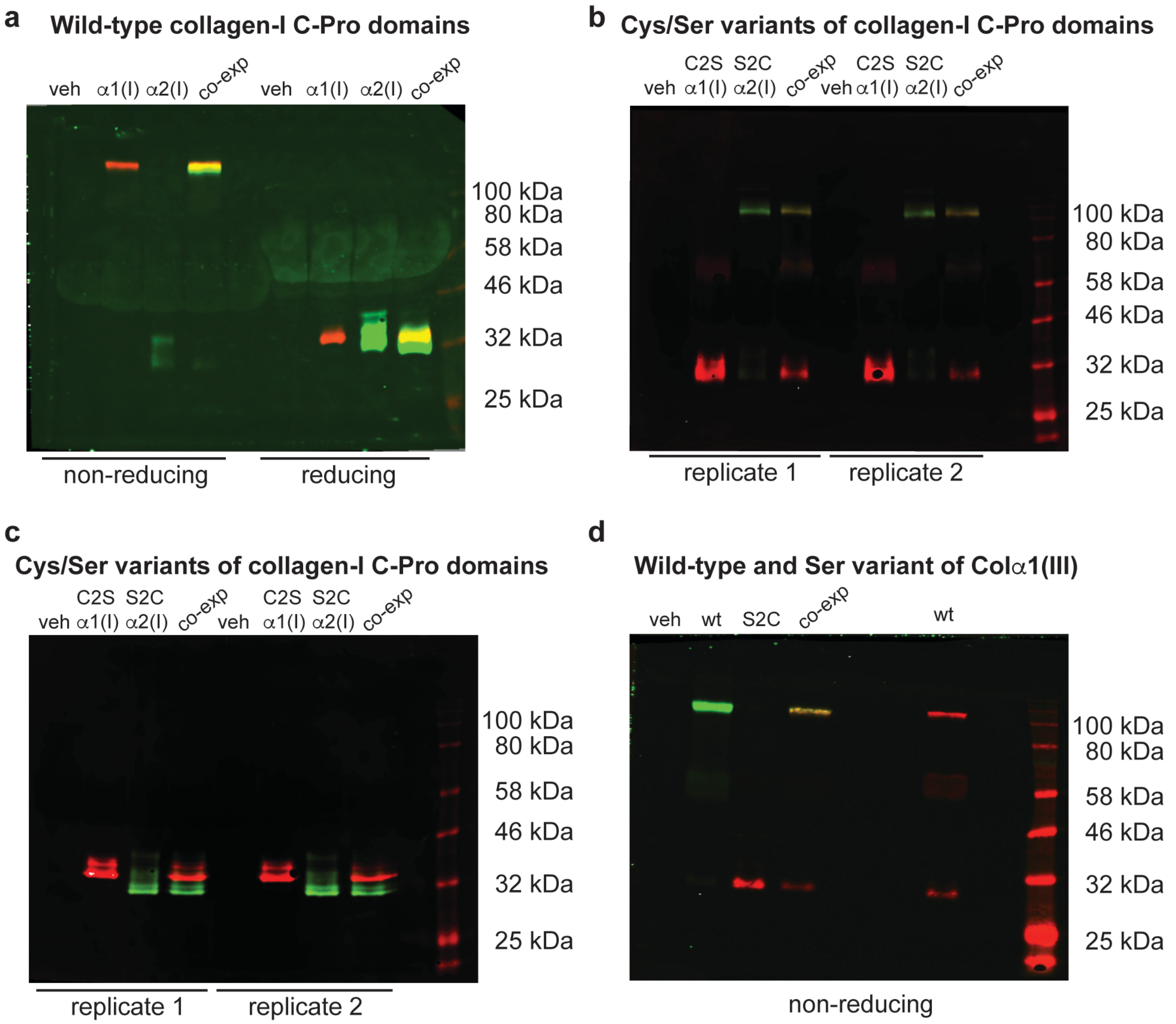

Supplementary Figure 7 | Uncropped immunoblots for select results

Select immunoblots are shown in the raw, uncropped form for transparency. The immunoblots shown correspond to Fig. 3a **(a)**, Fig. 3b **(b)**, Fig. 3c **(c)**, and Fig. 7d **(d)**.

SUPPLEMENTARY TABLES

Supplementary Table 1. Disulfide bonding network of wild-type C-Proα1(I).

Disulfide linkage	Digestion condition	Expected mass (charge)	Observed mass (charge)	Error (ppm)
C1 + C4	GluC + LysC	957.4489 (+2)	957.4457 (+2)	3.33
		638.6352 (+3)	638.6330 (+3)	3.44
		479.2284 (+4)	479.2269 (+4)	3.04
		383.5843 (+5)	383.5823 (+5)	5.08
C2 + C3	GluC + LysC	1327.5892 (+2)	1327.5896 (+2)	0.31
		885.3954 (+3)	885.3944 (+3)	1.13
		664.2985 (+4)	664.2977 (+4)	1.21
		531.6404 (+5)	531.6400 (+5)	0.70
C5 + C8	GluC + LysC	1310.6386 (+2)	1310.6331 (+2)	4.23
		874.0950 (+3)	874.0915 (+3)	4.04
		655.8232 (+4)	655.8195 (+4)	5.69
C6 + C7	GluC + LysC	1198.8800 (+3)	1198.8787 (+3)	1.11
		899.4120 (+4)	899.4109 (+4)	1.20
		719.7312 (+5)	719.7332 (+5)	2.85
		599.9440 (+6)	599.9439 (+6)	0.05
		514.3816 (+7)	514.3812 (+7)	0.83

Supplementary Table 2. Disulfide bonding network of S2C C-Proα2(I).

Disulfide linkage	Digestion condition	Expected mass (charge)	Observed mass (charge)	Error (ppm)
C1 + C4	GluC	724.0294 (+3)	724.0243 (+3)	7.00
	GluC	543.2740 (+4)	543.2710 (+4)	5.49
	GluC	434.8208 (+5)	434.8181 (+5)	6.09
C2 + C3	GluC	577.5895 (+3)	577.5933 (+3)	6.64
	GluC	433.4441 (+4)	433.4469 (+4)	6.56
C5 + C8	GluC	839.9301 (+2)	839.928 (+2)	2.49
	GluC	560.2893 (+3)	560.2884 (+3)	1.67
	GluC	420.4690 (+4)	420.4677 (+4)	2.99
C6 + C7	trypsin	1024.1642 (+3)	1024.1644 (+3)	0.23
	trypsin	768.3751 (+4)	768.3765 (+4)	1.85
	trypsin	614.9016 (+5)	614.9039 (+5)	3.69

Supplementary Table 3. Primers used for site-directed mutagenesis of the indicated C-Pro domains.

Primer Name	Sequence
COL1A1 C1265S F	5′- CCGTGACCTCAAGATGTCCCACTCTGACTGGAAG -3′
COL1A1 C1265S R	5′- CTTCCAGTCAGAGTGGGACATCTTGAGGTCACGG -3′
COL1A2 S1169C F	5′- CGTGACTTGAGACTCUGCCACCCAGAGTGGAG -3′
COL1A2 S1169C R	5′- CTCCACTCTGGGTGGCAGAGTCTCAAGTCACG -3′
COL2A1 C1306S F	5′-CCCCAACCAAGGCAGCACCTTGGACGC-3′
COL2A1 C1306S R	5′-GCGTCCAAGGTGCTGCCTTGGTTGGGG-3′
COL3A1 C1268S F	5′-TTGAGTTCAGGATGGCTGAATTTCAGGTCTCTGC-3′
COL3A1 C1268S R	5′-GCAGAGACCTGAAATTCAGCCATCCTGAACTCAA-3′
COL5A1 C1645S F	5′-AGTCGGGGTGGCTGAGCTGCAGGTC-3′
COL5A1 C1645S R	5′-GACCTGCAGCTCAGCCACCCCGACT-3′
COL5A2 S1319C F	5′-TTGATTGCATCTTCAACACATCCTTGGTTAGGATCAATCC-3′
COL5A2 S1319C R	5′-GGATTGATCCTAACCAAGGATGTGTTGAAGATGCAATCAA-3′
COL5A3 N1550C F	5′-CGAGCTGCACCGCTGCCACCCGCACCTG-3′
COL5A3 N1550C R	5′-CAGGTGCGGGTGGCAGCGGTGCAGCTCG-3′
COL11A1 S1613C F	5′-GGGAAGTCAGGATGGCAGAGTTGCAGGTCTTTA-3′
COL11A1 S1613C R	5′-TAAAGACCTGCAACTCTGCCATCCTGACTTCCC-3′
COL11A2 C1577S F	5′-AGCTCTGGGTGGCTCAGCTTCAGGTCC-3′
COL11A2 C1577S R	5′-GGACCTGAAGCTGAGCCACCCAGAGCT-3′
COL24A1 C1551S F	5′-TCCATCTGATACTTTTTGTTCACTGTTAAGTAAATCTTTGCAGATTC-3′
COL24A1 C1551S R	5′-GAATCTGCAAAGATTTACTTAACAGTGAACAAAAAGTATCAGATGGA-3′
COL27A1 C1696S F	5′-CCATCTTCTGCTCACTGTCCATGAGGTCCCT-3′
COL27A1 C1696S R	5′-AGGGACCTCATGGACAGTGAGCAGAAGATGG-3′

Supplementary Table 4. Molecular properties (calculated and measured) of collagen-I C-Pro variants.

	**wild-type C-Proα1(I)**	**Cys1265Ser C-Proα1(I)**	**wild-type C-Proα2(I)**	**Ser1169Cys C-Proα2(I)**
**residues**	251	251	252	252
M_S **(Da)**	27,909	27,893	28,225	28,241
$\bar{\nu}$ **(mL/g)**	0.723	0.723	0.728	0.728
ε **(M^{-1} cm^{-1})**	39,420	39,420	43,890	43,890
**MALDI-TOF (Da)**	29,183	29,174	29,371	29,476

SUPPLEMENTARY NOTE 1

Sequences of Open Reading Frames for C-Pro Domains

Color legend for all plasmids: Pre-protrypsin leader sequence Epitope tag HRV-3C protease cleavage site C-Pro domain

Wild-type PPT.His.C-Proα1(I):

ATGTCTGCACTTCTGATCCTAGCTCTTGTTGGAGCTGCAGTTGCTTACCCATACGATGTTCCAGATTACGC
TCACCATCACCATCACCATCTTGAAGTTCTTTTTCAAGGTCCTGATGATGCCAATGTGGTTCGTGACCGTG
ACCTCGAGGTGGACACCACCCTCAAGAGCCTGAGCCAGCAGATCGAGAACATCCGGAGCCCAGAGGGC
AGCCGCAAGAACCCCGCCCGCACCTGCCGTGACCTCAAGATGTGCCACTCTGACTGGAAGAGTGGAGAG
TACTGGATTGACCCCAACCAAGGCTGCAACCTGGATGCCATCAAAGTCTTCTGCAACATGGAGACTGGTG
AGACCTGCGTGTACCCCACTCAGCCCAGTGTGGCCCAGAAGAACTGGTACATCAGCAAGAACCCCAAGG
ACAAGAGGCATGTCTGGTTCGGCGAGAGCATGACCGATGGATTCCAGTTCGAGTATGGCGGCCAGGGCT
CCGACCCTGCCGATGTGGCCATCCAGCTGACCTTCCTGCGCCTGATGTCCACCGAGGCCTCCCAGAACA
TCACCTACCACTGCAAGAACAGCGTGGCCTACATGGACCAGCAGACTGGCAACCTCAAGAAGGCCCTGC
TCCTCCAGGGCTCCAACGAGATCGAGATCCGCGCCGAGGGCAACAGCCGCTTCACCTACAGCGTCACTG
TCGATGGCTGCACGAGTCACACCGGAGCCTGGGGCAAGACAGTGATTGAATACAAAACCACCAAGACCT
CCCGCCTGCCCATCATCGATGTGGCCCCCTTGGACGTTGGTGCCCCAGACCAGGAATTCGGCTTCGACG
TTGGCCCTGTCTGCTTCCTGTAA

Wild-type PPT.His.C-Proα2(I):

ATGTCTGCACTTCTGATCCTAGCTCTTGTTGGAGCTGCAGTTGCTGACTACAAAGACGATGACGACAAGC
ACCATCACCATCACCATCTTGAAGTTCTTTTTCAAGGTCCTGCTGACCAGCCTCGCTCAGCACCTTCTCTC
AGACCCAAGGACTATGAAGTTGATGCTACTCTGAAGTCTCTCAACAACCAGATTGAGACCCTTCTTACTCC
TGAAGGCTCTAGAAAGAACCCAGCTCGCACATGCCGTGACTTGAGACTCAGCCACCCAGAGTGGAGCAG
TGGTTACTACTGGATTGACCCTAACCAAGGATGCACTATGGATGCTATCAAAGTATACTGTGATTTCTCTA
CTGGCGAAACCTGTATCCGGGCCCAACCTGAAAACATCCCAGCCAAGAACTGGTATAGGAGCTCCAAGG
ACAAGAAACACGTCTGGCTAGGAGAAACTATCAATGCTGGCAGCCAGTTTGAATATAATGTAGAAGGAGT
GACTTCCAAGGAAATGGCTACCCAACTTGCCTTCATGCGCCTGCTGGCCAACTATGCCTCTCAGAACATC
ACCTACCACTGCAAGAACAGCATTGCATACATGGATGAGGAGACTGGCAACCTGAAAAAGGCTGTCATTC
TACAGGGCTCTAATGATGTTGAACTTGTTGCTGAGGGCAACAGCAGGTTCACTTACACTGTTCTTGTAGAT
GGCTGCTCTAAAAAGACAAATGAATGGGGAAAGACAATCATTGAATACAAAACAAATAAGCCATCACGCCT
GCCCTTCCTTGATATTGCACCTTTGGACATCGGTGGTGCTGACCAGGAATTCTTTGTGGACATTGGCCCA
GTCTGTTTCAAATAA

PPT.HA C-Proα1(II):

ATGTCTGCACTTCTGATCCTAGCTCTTGTTGGAGCTGCAGTTGCTTACCCATACGATGTTCCAGATTACGC
TGCGGCCGCAGACCAGGCAGCCGGTGGCCTGAGACAGCATGACGCCGAGGTGGATGCCACACTCAAGT
CCCTCAACAACCAGATTGAGAGCATCCGCAGCCCCGAGGGCTCCCGCAAGAACCCTGCTCGCACCTGCA
GAGACCTGAAACTCTGCCACCCTGAGTGGAAGAGTGGAGACTACTGGATTGACCCCAACCAAGGCTGCA
CCTTGGACGCCATGAAGGTTTTCTGCAACATGGAGACTGGCGAGACTTGCGTCTACCCCAATCCAGCAAA
CGTTCCCAAGAAGAACTGGTGGAGCAGCAAGAGCAAGGAGAAGAAACACATCTGGTTTGGAGAAACCAT
CAATGGTGGCTTCCATTTCAGCTATGGAGATGACAATCTGGCTCCCAACACTGCCAACGTCCAGATGACC
TTCCTACGCCTGCTGTCCACGGAAGGCTCCCAGAACATCACCTACCACTGCAAGAACAGCATTGCCTATC
TGGACGAAGCAGCTGGCAACCTCAAGAAGGCCCTGCTCATCCAGGGCTCCAATGACGTGGAGATCCGGG
CAGAGGGCAATAGCAGGTTCACGTACACTGCCCTGAAGGATGGCTGCACGAAACATACCGGTAAGTGGG

GCAAGACTGTTATCGAGTACCGGTCACAGAAGACCTCACGCCTCCCCATCATTGACATTGCACCCATGGA
CATAGGAGGGCCCGAGCAGGAATTCGGTGTGGACATAGGGCCGGTCTGCTTCTTGTAA

PPT.HA C-Proα1(III):

ATGTCTGCACTTCTGATCCTAGCTCTTGTTGGAGCTGCAGTTGCTTACCCATACGATGTTCCAGATTACGC
TGCGGCCGCAGATGAACCAATGGATTTCAAAATCAACACCGATGAGATTATGACTTCACTCAAGTCTGTTA
ATGGACAAATAGAAAGCCTCATTAGTCCTGATGGTTCTCGTAAAAACCCCGCTAGAAACTGCAGAGACCT
GAAATTCTGCCATCCTGAACTCAAGAGTGGAGAATACTGGGTTGACCCTAACCAAGGATGCAAATTGGAT
GCTATCAAGGTATTCTGTAATATGGAAACTGGGGAAACATGCATAAGTGCCAATCCTTTGAATGTTCCACG
GAAACACTGGTGGACAGATTCTAGTGCTGAGAAGAAACACGTTTGGTTTGGAGAGTCCATGGATGGTGGT
TTTCAGTTTAGCTACGGCAATCCTGAACTTCCTGAAGATGTCCTTGATGTGCAGCTGGCATTCCTTCGACT
TCTCTCCAGCCGAGCTTCCCAGAACATCACATATCACTGCAAAAATAGCATTGCATACATGGATCAGGCCA
GTGGAAATGTAAAGAAGGCCCTGAAGCTGATGGGGTCAAATGAAGGTGAATTCAAGGCTGAAGGAAATAG
CAAATTCACCTACACAGTTCTGGAGGATGGTTGCACGAAACACACTGGGGAATGGAGCAAAACAGTCTTT
GAATATCGAACACGCAAGGCTGTGAGACTACCTATTGTAGATATTGCACCCTATGACATTGGTGGTCCTGA
TCAAGAATTTGGTGTGGACGTTGGCCCTGTTTGCTTTTTATAA

PPT.HA C-Proα1(V):

ATGTCTGCACTTCTGATCCTAGCTCTTGTTGGAGCTGCAGTTGCTTACCCATACGATGTTCCAGATTACGC
TGCGGCCGCAGACGGCATGGAAGAGATCTTCGGCTCTCTCAACTCTCTGAAGCTGGAGATTGAGCAGAT
GAAACGGCCCCTGGGCACGCAGCAGAACCCCGCCCGCACCTGCAAGGACCTGCAGCTCTGCCACCCCG
ACTTCCCAGATGGTGAATACTGGGTCGATCCTAACCAAGGATGCTCCAGGGATTCCTTCAAGGTTTACTG
CAACTTCACAGCCGGGGGGTCGACATGCGTCTTCCCTGACAAGAAGTCCGAAGGGGCCAGAATCACTTC
TTGGCCCAAAGAAAACCCGGGCTCCTGGTTCAGTGAATTCAAGCGTGGGAAACTGCTCTCCTATGTGGAC
GCCGAGGGCAACCCTGTGGGTGTGGTACAGATGACCTTCCTGCGGCTGCTGAGCGCCTCTGCCCACCA
GAACGTCACCTACCACTGCTACCAGTCAGTGGCCTGGCAGGACGCAGCCACGGGCAGCTACGACAAGG
CCCTCCGCTTCCTGGGCTCCAACGACGAGGAGATGTCCTATGACAACAACCCCTACATCCGCGCCCTGG
TGGACGGCTGTGCTACCAAGAAAGGCTACCAGAAGACGGTTCTGGAGATCGACACCCCCAAAGTGGAGC
AGGTGCCCATCGTGGACATCATGTTCAATGACTTCGGTGAAGCGTCACAGAAATTTGGATTTGAAGTGGG
GCCGGCTTGCTTCATGGGCTAG

PPT.HA C-Proα2(V):

ATGTCTGCACTTCTGATCCTAGCTCTTGTTGGAGCTGCAGTTGCTTACCCATACGATGTTCCAGATTACGC
TGCGGCCGCAACAGCTGCTCTTGGGGATATCATGGGGCACTATGATGAAAGCATGCCAGATCCACTTCCT
GAGTTTACTGAAGATCAGGCGGCTCCTGATGACAAAAACAAAACGGACCCAGGGGTTCATGCTACCCTGA
AGTCACTCAGTAGTCAGATTGAAACCATGCGCAGCCCCGATGGCTCGAAAAAGCACCCAGCCCGCACGT
GTGATGACCTAAAGCTTTGCCATTCCGCAAAGCAGAGTGGTGAATACTGGATTGATCCTAACCAAGGATCT
GTTGAAGATGCAATCAAAGTTTACTGCAACATGGAAACAGGAGAAACATGTATTTCAGCAAACCCATCCAG
TGTACCACGTAAAACCTGGTGGGCCAGTAAATCTCCTGACAATAAACCTGTTTGGTATGGTCTTGATATGA
ACAGAGGGTCTCAGTTCGCTTATGGAGACCACCAATCACCTAATACAGCCATTACTCAGATGACTTTTTTG
CGCCTTTTATCAAAAGAAGCCTCCCAGAACATCACTTACATCTGTAAAAACAGTGTAGGATACATGGACGA
TCAAGCTAAGAACCTCAAAAAAGCTGTGGTTCTCAAAGGGGCAAATGACTTAGATATCAAAGCAGAGGGA
AATATTAGATTCCGGTATATCGTTCTTCAAGACACTTGCTCTAAGCGGAATGGAAATGTGGGCAAGACTGT
CTTTGAATATAGAACACAGAATGTGGCACGCTTGCCCATCATAGATCTTGCTCCTGTGGATGTTGGCGGC
ACAGACCAGGAATTCGGCGTTGAAATTGGGCCAGTTTGTTTTGTGTAA

PPT.HA C-Proα3(V):

ATGTCTGCACTTCTGATCCTAGCTCTTGTTGGAGCTGCAGTTGCTTACCCATACGATGTTCCAGATTACGC
TGCGGCCGCAGAGGAGGTGCTGGCCTCGCTCACATCGCTGAGCTTGGAGCTGGAGCAGCTGCGGCGTC
CTCCCGGCACTGCGGAGCGCCCGGGCCTCGTGTGCCACGAGCTGCACCGCAACCACCCGCACCTGCCT
GATGGGGAATACTGGATTGACCCCAACCAGGGCTGCGCGCGGGACTCGTTCAGGGTTTTTTGCAACTTC
ACGGCGGGAGGAGAGACCTGCCTCTATCCCGACAAGAAGTTTGAGATCGTGAAATTGGCCTCCTGGTCC
AAGGAAAAGCCTGGAGGCTGGTATAGCACATTCCGTCGAGGGAAGAAGTTCTCCTACGTGGACGCCGAC
GGGTCCCCAGTGAATGTCGTGCAGCTGAACTTCCTGAAACTGCTGAGTGCCACAGCTCGCCAGAACTTCA
CCTACTCCTGCCAGAATGCAGCTGCCTGGCTGGACGAAGCCACGGGTGACTACAGCCACTCCGCCCGCT
TCCTTGGCACCAATGGAGAGGAGCTGTCTTTCAACCAGACGACAGCAGCCACTGTCAGCGTCCCCCAGG
ATGGCTGCCGGCTCCGGAAAGGACAGACGAAGACCCTTTTCGAATTCAGCTCTTCTCGAGCGGGATTTCT
GCCCCTGTGGGATGTGGCGGCCACTGACTTTGGCCAGACGAACCAAAAGTTTGGGTTTGAACTGGGCCC
CGTCTGCTTCAGCAGCTGA

PPT.HA C-Proα1(XI):

ATGTCTGCACTTCTGATCCTAGCTCTTGTTGGAGCTGCAGTTGCTTACCCATACGATGTTCCAGATTACGC
TGCGGCCGCAGATGCAGATGATAATATTCTTGATTACTCGGATGGAATGGAAGAAATATTTGGTTCCCTCA
ATTCCCTGAAACAAGACATTGAGCATATGAAATTTCCAATGGGTACTCAGACCAATCCAGCCCGAACTTGT
AAAGACCTGCAACTCAGCCATCCTGACTTCCCAGATGGTGAATATTGGATTGATCCTAACCAAGGTTGCTC
AGGAGATTCCTTCAAAGTTTACTGTAATTTCACATCTGGTGGTGAGACTTGCATTTATCCAGACAAAAAATC
TGAGGGAGTAAGAATTTCATCATGGCCAAAGGAGAAACCAGGAAGTTGGTTTAGTGAATTTAAGAGGGGA
AAACTGCTTTCATACTTAGATGTTGAAGGAAATTCCATCAATATGGTGCAAATGACATTCCTGAAACTTCTG
ACTGCCTCTGCTCGGCAAAATTTCACCTACCACTGTCATCAGTCAGCAGCCTGGTATGATGTGTCATCAG
GAAGTTATGACAAAGCACTTCGCTTCCTGGGATCAAATGATGAGGAGATGTCCTATGACAATAATCCTTTT
ATCAAAACACTGTATGATGGTTGTGCGTCCAGAAAAGGCTATGAAAAGACTGTCATTGAAATCAATACACC
AAAAATTGATCAAGTACCTATTGTTGATGTCATGATCAATGACTTTGGTGATCAGAATCAGAAGTTCGGATT
TGAAGTTGGTCCTGTTTGTTTTCTTGGCTAA

PPT.HA C-Proα2(XI):

ATGTCTGCACTTCTGATCCTAGCTCTTGTTGGAGCTGCAGTTGCTTACCCATACGATGTTCCAGATTACGC
TGCGGCCGCAATCCAGCCACTGCCCATTCAGATGCCCAAGAAGACTCGGCGCTCGGTGGATGGAAGCCG
TCTGATGCAGGAAGATGAGGCCATACCGACCGGGGGAGCCCCCGGCAGTCCTGGGGGGCTGGAGGAGA
TCTTTGGCTCACTCGACTCCCTGCGGGAGGAGATCGAGCAGATGAGGCGGCCAACAGGGACCCAGGAC
AGCCCTGCTCGCACCTGCCAGGACCTGAAGCTGTGCCACCCAGAGCTTCCCGATGGAGAGTACTGGGTC
GACCCCAACCAGGGCTGTGCTCGGGATGCCTTCCGAGTTTTCTGCAACTTCACAGCAGGGGGTGAGACC
TGTGTGACGCCTAGGGATGACGTCACGCAGTTCTCTTACGTGGACTCAGAGGGCTCCCCAGTGGGTGTG
GTCCAGCTCACCTTCCTGCGGCTGCTCAGCGTCTCAGCCCACCAGGACGTCTCCTACCCCTGCTCTGGA
GCAGCCCGTGACGGTCCCCTGAGACTCCGTGGGGCCAATGAGGATGAGCTGAGCCCGGAGACTAGCCC
CTATGTCAAAGAATTCAGAGATGGCTGCCAGACACAGCAAGGCCGGACGGTGCTGGAGGTGCGAACGCC
TGTGCTGGAGCAGCTGCCAGTGCTGGATGCCTCCTTCTCAGACCTGGGAGCCCCACCGAGGCGGGGAG
GGGTGCTGCTGGGGCCTGTCTGCTTCATGGGATAG

PPT.HA C-Proα1(XXIV):

ATGTCTGCACTTCTGATCCTAGCTCTTGTTGGAGCTGCAGTTGCTTACCCATACGATGTTCCAGATTACGC
TGCGGCCGCAGAAGAGATATTCAAAACCCTGAACTACCTTAGCAATTTATTGCACAGCATCAAGAATCCTC
TTGGCACACGAGATAACCCAGCACGAATCTGCAAAGATTTACTTAACTGTGAACAAAAAGTATCAGATGGA
AAATACTGGATTGACCCAAATCTTGGCTGTCCTTCAGATGCCATTGAGGTTTTCTGCAATTTCAGTGCTGG

TGGCCAGACATGCTTACCTCCTGTTTCTGTAACAAAGTTGGAGTTTGGAGTTGGGAAAGTCCAGATGAACT
TCCTTCATTTACTGAGTTCGGAAGCCACCCATATCATCACCATTCACTGTCTAAACACCCCAAGGTGGACA
AGCACACAAACAAGTGGCCCAGGATTGCCTATTGGTTTCAAGGGATGGAATGGCCAGATTTTTAAAGTAAA
CACTCTACTTGAACCTAAAGTGCTTTCAGATGACTGCAAGATTCAAGATGGCAGCTGGCATAAGGCAACAT
TTCTTTTTCACACCCAGGAACCTAATCAACTTCCAGTGATTGAAGTACAAAAACTTCCTCATCTCAAAACTG
AACGAAAGTATTACATTGACAGCAGTTCTGTATGCTTTCTGTAA

PPT.HA C-Proα1(XXVII):

ATGTCTGCACTTCTGATCCTAGCTCTTGTTGGAGCTGCAGTTGCTTACCCATACGATGTTCCAGATTACGC
TGCGGCCGCAATCCAATTGCAACAAGATGATCTTGGGGCAGCTTTCCAGACGTGGATGGACACCAGTGG
AGCACTCAGGCCAGAGAGTTACAGCTATCCAGACCGGCTGGTGCTGGACCAGGGAGGAGAGATCTTTAA
AACCTTACACTACCTCAGCAACCTCATCCAGAGCATTAAGACGCCCCTGGGCACCAAAGAGAACCCCGCC
CGGGTCTGCAGGGACCTCATGGACTGTGAGCAGAAGATGGTGGATGGTACCTACTGGGTGGATCCAAAC
CTTGGCTGCTCCTCTGACACCATCGAGGTCTCCTGCAACTTCACTCATGGTGGACAGACGTGTCTCAAGC
CCATCACGGCCTCCAAGGTCGAGTTTGCCATCAGCCGGGTCCAGATGAATTTCCTGCACCTGCTAAGCTC
CGAGGTGACCCAGCACATCACCATCCACTGCCTTAACATGACCGTGTGGCAGGAGGGCACTGGGCAGAC
CCCAGCCAAGCAGGCCGTACGCTTCCGGGCCTGGAATGGACAGATTTTTGAAGCTGGGGGTCAGTTCCG
GCCCGAGGTGTCCATGGATGGCTGCAAGGTCCAAGATGGCCGCTGGCATCAGACACTCTTCACCTTCCG
GACCCAAGACCCCCAACAGCTGCCCATCATCAGTGTGGACAACCTCCCTCCTGCCTCATCAGGGAAGCA
GTACCGCCTGGAAGTTGGACCTGCGTGCTTCCTCTGA
